# Ag(II) as Spin Super-Polarizer in Molecular Spin Clusters

**DOI:** 10.1021/acs.jpca.2c06032

**Published:** 2022-12-15

**Authors:** Mateusz Domański, Jan van Leusen, Marvin Metzelaars, Paul Kögerler, Wojciech Grochala

**Affiliations:** † Center of New Technologies, University of Warsaw, Zwirki i Wigury 93, 02089Warsaw, Poland; ‡ Institut für Anorganische Chemie, RWTH Aachen University, 52056Aachen, Germany

## Abstract

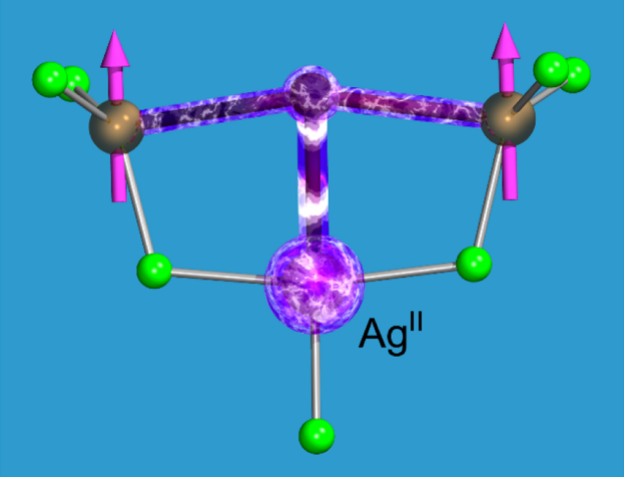

Using quantum mechanical calculations, we examine magnetic (super)exchange interactions in hypothetical, chemically reasonable molecular coordination clusters containing fluoride-bridged late transition metals or selected lanthanides, as well as Ag(II). By referencing to analogous species comprising closed-shell Cd(II), we provide theoretical evidence that the presence of Ag(II) may modify the magnetic properties of such systems (including metal–metal superexchange) to a surprising degree, specifically both coupling sign and strength may markedly change. Remarkably, this happens in spite of the fact that the fluoride ligand is the least susceptible to spin polarization among all monoatomic ligands known in chemistry. In an extreme case of an oxo-bridged Ni(II)_2_ complex, the presence of Ag(II) leads to a nearly 17-fold increase of magnetic superexchange and switching from antiferro (AFM)- to ferromagnetic (FM) coupling. Ag(II)—with one hole in its d shell that may be shared with or transferred to ligands—effectively acts as spin super-polarizer, and this feature could be exploited in spintronics and diverse molecular devices.

## Introduction

Spintronics is one of the widely developing fields of modern science and technology.^1−7^ This vast subdiscipline of nanotechnology, drawing from physics, chemistry, and materials science, relies on the manipulation of individual spins either in the solid state or in small molecular assemblies using light and electric and/or magnetic fields. In this context, controllable and precisely engineered molecular devices are expected to revolutionize information processing in terms of density, speed, and energy consumption. Realizing key spintronics functionalities on a molecular scale commonly mandates high magnetic anisotropy and robust coupling, mirroring the merits that have been strived for over the past 2 decades in the development of single-molecule magnets that feature either transition metals (TM, d-block elements), lanthanides (Ln, f-block elements), or both.^8−13^ While Ln-based polynuclear clusters may exhibit many high multiplet states and pronounced anisotropy, the inherently weak^14^ Ln–Ln magnetic interactions limit the prospects for exchange-coupled high-spin (HS) systems. On the other hand, clusters containing TMs can assume fewer quantum states but the characteristics of their magnetically relevant orbitals lead to significantly higher coupling energies. One compromise at hand is to construct polynuclear clusters containing both TM and Ln centers. Still, there is a pressing need to manipulate and greatly increase the magnetic coupling interactions present in such systems.^15−22^

Two families of compounds stand out among the materials exhibiting the strongest magnetic interactions known to date: oxocuprates(II) and fluoroargentates(II) (see Table 1). Magnetic superexchange interactions via a single O (or, respectively, F) bridge range up to −260 meV (−2097 cm^–1^) in the former and up to −331 meV (−2670 cm^–1^) for the latter, but they are consistently strong in quasi-molecular (0D), one-dimensional, and two-dimensional systems. Substantial mixing of the metal d with the nonmetal p valence states (“covalence”) is one reason for these materials’ uniqueness.^23−27^

**Table 1:** Selection of the Compounds Featuring Giant Absolute Values of the Magnetic Superexchange Constant between Two Identical Metal Centers, Quoting the Largest Values Given in the Literature for Each Compound^a^

formula	superexchange pathway	*J*/meV^b^	magnetic dimensionality	references
La_2_CuO_4_	Cu–O–Cu	–153	2D	(36)
Sr_2_CuO_2_Cl_2_	Cu–O–Cu	–130	2D	(37)
Sr_2_CuO_3_	Cu–O–Cu	–260	1D	(38)
AgF_2_	Ag–F–Ag	–70	2D	(39)
[AgF_2_]^c^	Ag–F–Ag	–265^e^	2D	(40)
HT-KAgF_3_	Ag–F–Ag	–100	1D	(41)
LT-KAgF_3_	Ag–F–Ag	–113^e^	1D	(42, 43)
RbAgF_3_	Ag–F–Ag	–144^e^	1D	(42, 43)
CsAgF_3_	Ag–F–Ag	–161^e^	1D	(42, 43)
AgFBF_4_	Ag–F–Ag	–331^e^	1D	(42, 43)
Ag_2_ZnZr_2_F_14_	Ag–F–Ag	–313^e^	0D	(44)
AgF_2_-HP2^d^	Ag–F–Ag	–250^e^	0D	(44)
Rb_3_Ag_2_F_7_	Ag–F–Ag	–240^e^	0D	(45)

a Magnetic dimensionality indicates in how many directions the said interaction is operational.

b Using a Heisenberg–Dirac–van Vleck Hamiltonian for two interacting spin centers of the type  with (*J*_*ij*_ = *J*_*ji*_); consequently, a negative *J* value indicates AFM interaction.

c Single layer deposited on RbMgF_3_ substrate.

d High-pressure structure type 2.

e Theoretical value.

It might be anticipated that these two late d-shell metal cations may induce a strong electron correlation in systems comprising TM or Ln cations. Indeed, copper clusters were already extensively studied in the context of mediating or inducing magnetic exchange with Ln cations, focusing on Cu–Ln exchange.^28−30^ Such Cu–Ln interaction usually is much stronger than that for pure Ln–Ln, and even for a short distance present for the Ln–(μ_2_-O)_2_–Ln bridge it is well below 1 cm^–1^, for example, −0.178 cm^–1^ for a typical Dy(III) complex.^31^ Usually, the coupling energy *J* associated with a Ln–(μ_2_-O)_2_–Cu bridge is FM, ranging from ∼0 up to +13 cm^–1^.^28−30^ The corresponding Ag(II) systems have not yet been studied in experiment or theoretically. Nonetheless, Ag(II) is exceptional among the group 11 element cations, as here spin polarization may strongly affect even the attached fluoride anion. Although this ligand is not capable of transmitting strong magnetic superexchange^32−34^ (as fluorine is the least spin-polarizable monoatomic ligand species known in chemistry), the presence of Ag(II) effectively enforces such an interaction^35^ even in fluorides. Thus, the possibility of introducing Ag(II) to Ln or TM-based fluoride core structures as single-molecular magnet moieties is enticing.

For instance, in FM systems containing AgF_4_^2–^ anions, as much as half of the whole spin density may be located on four fluoride ligands, while the remainder resides on the Ag(II) center.^46^ Such unusual properties allow us to treat both Cu(II) oxides and Ag(II) fluorides as redox non-innocent ligand systems. This motivates using Ag(II) as a spin polarizer toward a nonmetal ligand, which bridges two other magnetic metals (TM or Ln) and alters their magnetic superexchange in this way. This should be a particularly promising approach if the said bridging ligand could carry a net spin and as such would constitute an equivalent of an organic free radical. We note that superexchange is well known to become very strong if the bridging ligand has free-radical character.^47−49^ Apart from fluoride systems, which benefit from thermodynamic stability in the case of Ag(II),^25^ we can also envisage the presence of Ag(II) in an oxide system. Ag(II) compounds bearing monoatomic oxide anions are not known, but those with more complex oxoanions (sulfates, fluorosulfates, triflates, difluorophosphonates etc.) have been prepared. One argument for looking at oxide systems is that the spin–spin interactions might be transferred even easier than for fluoride due to more substantial spin polarization of the ligand. A similar concept could be extended to other anions more prone to oxidation than fluoride, for example, chloride, thus effectively increasing the ligand’s spin and strengthening the superexchange coupling.

Herein, we theoretically study the influence of the strong spin-polarizer, Ag(II), on magnetic interactions of diverse open-shell cations’ including late TMs and Lns. Specifically, we examine magnetic (super)exchange interactions in hypothetical, yet chemically reasonable molecular coordination clusters containing fluoride-bridged bimetallic systems and Ag(II). By referencing analogous species comprising closed-shell Cd(II), we provide theoretical evidence that the presence of Ag(II) may modify the magnetic properties of such systems (including metal–metal superexchange) to a marked degree, where both coupling sign and strength may change.

## Methods

Recently, numerous computational studies have centered on exchange coupling in molecular systems containing one or more Ln cations, for example, cerium,^50^ praseodymium,^51^ europium,^31^ gadolinium,^31^ terbium,^31,52^ dysprosium,^31,53^ or holmium.^31,53^ The pronounced effort in theoretical research on magnetic clusters containing Lns is connected to a correct description of 4f semi-core electrons. These levels are subject to a splitting originating from the ligand (crystal) field and spin–orbit interactions.^54^ In many of these studies,^31,50−53^ the spin–orbit coupling effects have been included. However, for most gadolinium studies^29,55−61^ where the spin–orbit coupling effect is predicted to be weak, the scalar-relativistic methods and the Ising-type Hamiltonian were used. One important issue with spin–orbit coupling is that it can be relatively easily employed for single-center systems, and usually a CAS approach is used for this. However, for larger systems, this approach becomes often too expensive, though the necessity persists to correctly account for relativistic effects.^29^ Some authors have suggested that in Ln systems, much more important than including spin–orbit coupling is assuring the correct ground state (GS) electronic configuration (which otherwise could induce differential correlation effects and would not systematically improve the calculated results).^62^

Due to the extensive range of hypothetical molecules considered in this study, we chose the hybrid density functional theory approach using the B3LYP functional^63,64^ that is frequently used in similar studies, together with a scalar relativistic Hamiltonian ZORA and all-electron basis set SARC-ZORA-TZVP for Ag and 4f metals and ZORA-def2-TZVP basis for the lighter elements.^62,65^ The exchange spin coupling parameters *J* were obtained using the “broken symmetry” (BS) formalism^66^ by calculating all possible spin states in each system and solving the set of linear equations. For this type of molecules, it was shown that hybrid functionals may overestimate the experimentally obtained coupling constants, however to a small degree.^29^

To validate this methodology, we have cross-checked our calculations with several examples from the literature where either experimental or high-level theoretical parameters are known. We have obtained satisfying results for both Cu–Ln and Ln–Ln superexchange interactions (see Supporting Information and Table S1). As we experienced, differences in electron orbital configurations of cations in excited states would induce enormous errors for magnetic superexchange interactions; thus, a great effort has been put into assuring that this is not the case here (see Tables S7–S12). More details of the computational methods are presented in the Supporting Information. Importantly, since our study focuses on a comparison between Ag(II) and related Cd(II) systems, a large share of methodological errors cancels out, thus showcasing the important trends more clearly. The use of Cd(II) systems as reference is dictated by similarity of ionic radii between Ag(II) and Cd(II) as well as the fact that Cd(II) is a closed-shell cation which does not permit facile transmission of the magnetic superexchange either.

## Results

**Figure 1:**
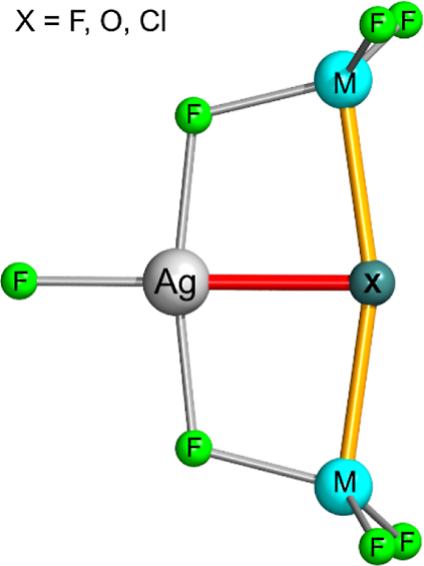
Schematic drawing of all optimized geometries of small metal–silver coordination clusters studied in this work. M–(μ-X)–M bonds are highlighted in orange, Ag–X bond in red. The reference systems feature d^10^ Cd^2+^ instead of d^9^ Ag^2+^. The stoichiometries are anionic [M_2_AgF_8_]^2–^ (M = Co, Ni, Cu), [M_2_AgF_7_Cl]^2–^ (M = Co, Ni), [M_2_AgF_7_O]^3–^ (M = Co, Ni, Cu), [M_2_AgF_7_O]^−^ (M = Sm, Eu, Gd, Tb, Dy, Ho, Er, Tm, Yb), neutral M_2_AgF_8_ (M = Nd, Pm, Sm), and M_2_AgF_7_Cl (M = Pr, Nd, Pm, Sm, Eu, Gd, Tb, Dy, Ho). M is divalent for Co, Ni, and Co, and trivalent for Lns.

We have tested the abovementioned overarching idea on triangular model clusters comprising a single Ag(II) and two other metal cations (Figure 1). This particular geometry type was chosen as it facilitates the influence of Ag(II) directly on the Ln–X–Ln bridge, which is the purpose of this work. Such bridges are common in the solid state, where a ligand bridging two larger cations is also coordinated to a smaller one, as present in, for example, the crystalline lattices of La_2_CuO_4_ or the K–Ag(II)–F series. Concurrently, the cluster geometry derives partly from the postulated Ln_2_F_6_ molecules^67−69^ and bridges present in some larger molecular magnets with Ln–(μ_2_-X)_2_–Ln bridges.^28−31^ Here,^32−34^ the AgF^+^ group is added, in a perpendicular orientation, to the center of the Ln–X–Ln bridge with other fluorides mainly stabilizing the central part. The chosen molecular clusters proved their stability in numerous geometry optimization runs and as such serve as a test bed for the spin–spin interactions.^35^

We note that the employed clusters including a quasi-triangular trimetallic core may be subject to geometrical spin-frustration. In particular, the AFM interactions between two metallic centers and Ag(II) may compete with the AFM coupling between two metal cations. Nevertheless, as will be shown below, most often the Ag–M interactions predominate and they determine the spin ordering of the GS.

Following our recent study we have chosen the three late 3d TMs Cu(II), HS Ni(II) and HS Co(II) since they offer a broad variety of spin moments from spin-1/2 to spin-3/2 and of metal–ligand bond covalence. Simultaneously, we have avoided using early TMs, which would be immediately oxidized by the Ag(II) cation.^25^ The bridging ligands, X, comprise a fluoride F^–^ (a ligand of choice in electron-deficient Ag(II) systems) and its isoelectronic siblings, O^2–^ and Cl^–^. For each M, the cluster geometry was optimized in its electronic GS configuration. To understand the impact of Ag(II) on the magnetic properties of the cluster and, in particular, on the M–M magnetic superexchange, we have referenced the results to those obtained for isostructural derivatives containing Cd instead of Ag. Cd(II) is a closed-shell diamagnetic cation incapable of transmitting magnetic superexchange to any significant degree and, therefore, the magnetic properties of Cd-bearing species are dominated by the contributions of the M–M superexchange mediated by the X bridge. Since the ionic radius of Cd(II) (Shannon–Prewitt provide the value of 1.09 Å for octahedral coordination) is very close to that of Ag(II) (1.08 Å for octahedral coordination) and in order to extract the impact of Ag(II) on electronic and magnetic properties free from strain effects, we kept the molecular geometry of each Cd-substituted species identical to that of its Ag(II) analogue.

**Figure 2:**
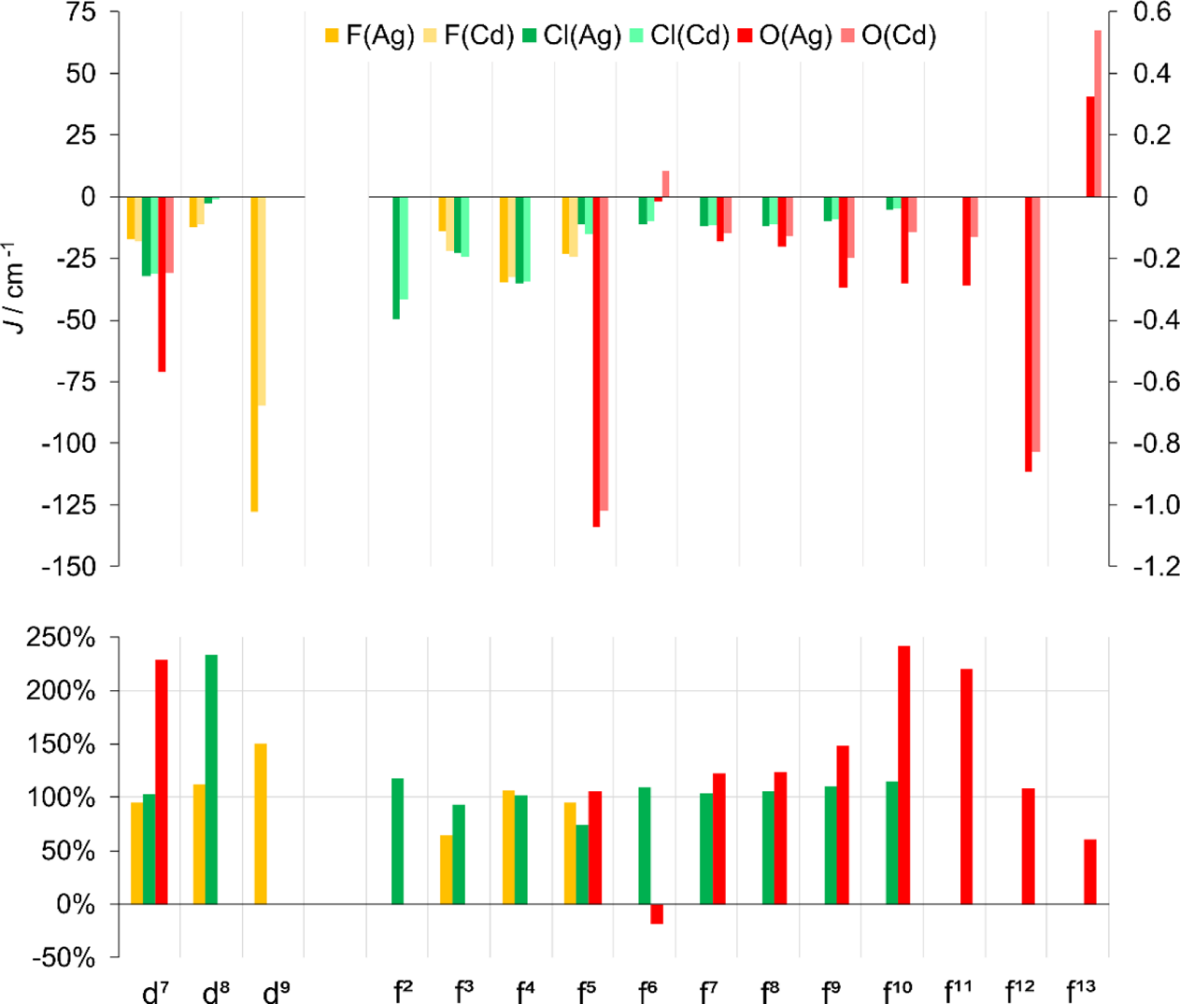
Impact of Ag(II) on metal–metal superexchange constant, *J*_MM_, as measured by (top) the values of *J*_MM_ and (bottom) the ratio (in %) of *J*_MM_ in the Ag(II)- vs Cd(II)-containing species, free from magnetostrictive effects. Here, +100% corresponds to no impact, whereas negative values imply a change in the interaction from AFM to FM or vice versa. *J* scales for TMs and Lns are separate. The results for the Ni_2_ cluster with central oxide ligands (cf. Table 2) are off the scale and have not been shown here.

In a first step, we explored the three aforementioned 3d TM cations and the entire series of partially filled f-subshell Ln cations in the given cluster geometry with the three central ligands, X: fluoride, and chloride or oxide as alternatives. We notice that, in some cases, a reorganization of the cluster geometry occurred, in particular with 3d TM cations, while some Ln clusters did not yield a stable arrangement. In other cases, a spontaneous redox reaction took place. For 3d TM cation clusters, the electronic and ionic stability was obtained for systems with fluoride bridging ligands for all cations, with chlorides for Co and Ni, and with oxide only for Ni cations. In the case of Ln cation clusters, we obtained electronic and ionic stability with the fluoride bridging ligand for f^3^–f^5^ cations, with chlorides for f^2^–f^10^ cations, and with oxide for f^5^–f^13^ cations. We, here, discuss all cases where the geometry scheme shown in Figure 1 represents a stable local minimum, only in two cases being associated with an excited spin state. The obtained changes of metal–metal superexchange interaction due to Ag(II) influence are presented in Figure 2, and numerical data are gathered in Table 2 (for further details see Figures S1, S2 and Table S2 in the Supporting Information). The resulting spin populations of the key atoms in the studied clusters are listed in Table S4.

**Table 2:** Computed Data for Each Considered System: Valence Electron Configurations (v.e.), GS Configurations, Values of Silver–Metal and Metal–Metal Superexchange Constants before and after Ag(II) → Cd(II) Substitution (*J*_AgM_, *J*_MM_(Ag), and *J*_MM_(Cd), Respectively) and Brief Analysis of These Values^a^

bridging ligand	metal	v.e.	GS	*J*_AgM_/cm^–1^	*J*_MM_(Ag)/cm^–1^	*J*_MM_(Cd)/cm^–1^	ratio (%)	Δ/cm^–1^
F^–^	Co	d^7^	BS(M)	41.0	–17.3	–18.2	95	0.9
	Ni	d^8^	BS(Ag)	–44.4	–12.5	–11.1	112	–1.4
	Cu	d^9^	BS(Ag)	–285.5	–127.6	–84.7	151	–42.9
	Nd	f^3^	BS(M)	–9.49	–0.11	–0.17	64	0.063
	Pm	f^4^	BS(Ag)	–1.78	–0.28	–0.26	106	–0.016
	Sm	f^5^	HS	2.69	–0.18	–0.19	95	0.009
O^2–^	Co	d^7^	HS*	326.5	–71.1	–31.1	229	–40.0
	Ni	d^8^	HS	409.1	1670.6	–100.5	–1662	1771.1
	Cu	d^9^	BS(M)*	–255.2	–608.7	–381.0	160	–227.7
	Sm	f^5^	BS(Ag)	–29.94	–1.073	–1.018	105	–0.055
	Eu	f^6^	BS(Ag)	–22.60	–0.016	0.084	–19	–0.100
	Gd	f^7^	BS(Ag)	–12.54	–0.146	–0.119	123	–0.027
	Tb	f^8^	BS(M)	–4.30	–0.160	–0.130	123	–0.030
	Dy	f^9^	BS(M)	–1.47	–0.293	–0.197	148	–0.096
	Ho	f^10^	HS	2.85	–0.281	–0.116	242	–0.164
	Er	f^11^	BS(Ag)	–8.99	–0.289	–0.131	220	–0.157
	Tm	f^12^	HS	21.71	–0.893	–0.826	108	–0.067
	Yb	f^13^	HS	19.61	0.326	0.540	60	–0.214
Cl^–^	Co	d^7^	BS(M)	7.7	–32.3	–31.5	103	–0.8
	Ni	d^8^	BS(Ag)	–83.1	–2.7	–1.1	234	–1.5
	Pr	f^2^	BS(Ag)	–31.44	–0.395	–0.334	118	–0.060
	Nd	f^3^	BS(M)	–4.48	–0.183	–0.196	93	0.013
	*P*m	f^4^	BS(Ag)	–1.95	–0.279	–0.275	101	–0.004
	Sm	f^5^	BS(M)	2.11	–0.090	–0.122	74	0.031
	Eu	f^6^	HS	2.91	–0.087	–0.080	109	–0.007
	Gd	f^7^	HS	2.11	–0.096	–0.092	104	–0.003
	Tb	f^8^	HS	4.93	–0.095	–0.090	105	–0.005
	Dy	f^9^	HS	6.88	–0.078	–0.071	111	–0.007
	Ho	f^10^	HS	1.79	–0.044	–0.038	115	–0.006

a Each system has one GS out of three possible spin-states: HS, broken-symmetry with S(Ag) of opposite polarity “BS(Ag)”, or broken-symmetry with S(M) of opposite polarity “BS(M)”. The ratio is defined as Ratio = *J*_MM_(Ag)/*J*_MM_(Cd), while the difference is Δ = *J*_MM_(Ag) – *J*_MM_(Cd). Clusters in principle may lack symmetry elements, thus *J*_AgM_ may be an average over two interactions (Ag–M^1^ and Ag–M^2^), *cf*. Supporting Information for details. Asterisks indicate that an electronic configuration, in which cluster geometry is stable, becomes metastable with respect to the other electronic configuration when relaxed (see more in the Supporting Information); however, such cases are rare.

Generally, smaller absolute effects are expected for Ln systems as compared to TM ones due to the semi-core nature of the f valence states. We note also that almost for each Ln system, the Ag–M interaction predominates the M–M one, being usually over an order of magnitude larger. Nevertheless, the introduction of Ag(II) in place of Cd(II) turned out to reveal some surprises. The effects for isotropic Gd(III) were rather small and up to +23%. On the other hand, the Eu f^6^ system with an O^2–^ bridge experiences a change from FM to AFM Ln–Ln interaction due to Ag^2+^ influence (in other words, the difference *J*_MM_(Ag) – *J*_MM_(Cd) is larger than the value of *J*_MM_(Cd) itself). The largest influences in terms of absolute values are obtained for Ho and Yb clusters, reaching up to 0.21 cm^–1^ for the former, which is equivalent to a 242% change relative to the Cd(II) reference cluster. Further enhancement can be anticipated to arise from proper engineering of the superexchange pathway and modifying the bridging ligands from fluoride to more spin-polarizable softer ligands.

A comparison of the *J* values for all molecules studied shows that in most cases the GS corresponds to an AFM interaction between the M centers, consistent with a nearly linear M–X–M bridge geometry. However, the Ag–M interactions vary both qualitatively (AFM vs FM) and quantitatively (i.e., from weak to strong) depending on the selected molecular system. This originates from differences in chemical bonding, geometry of the superexchange pathway for the two lateral fluoride bridges (with the M–F–Ag angle not far from 100°, which represents the border between FM and AFM superexchange^44^), as well as the shape of spin density at M cations that depends on the auxiliary ligand field. Interestingly and counterintuitively, the M–M AFM interaction is sometimes enhanced in the presence of Ag(II) as compared to Cd(II) by a factor of up to 2.5. This is, for example, the case for M = Cu in clusters with X = F or O. In this particular case, the spin density on the central ligand switches its polarization from the p orbital, which is more or less parallel to the Cu–Cu vector, to the one which is close to a perpendicular one. Still, the fact that the Cu–X–Cu bridge is not perfectly linear permits spin interaction to take place rather than to cancel out. Thus, the influence of Ag(II) is in principle more complex than initially assumed and may propagate via both a central and two auxiliary bridges.

The most interesting system seems to be the Ni_2_(μ-O) cluster, where the Ni–Ni superexchange changes dramatically from an initially strong AFM for Cd(II) (−100 cm^–1^) to a very strong FM for Ag(II) (+1670 cm^–1^). The geometry and spin density in this complex (see Supporting Information) reveal that this system best exemplifies the success of the approach postulated here. First, the Ni–O and Ag–O bonds are very short, thus facilitating strong interactions. Second, Ag(II) introduces a huge spin density amounting to 0.49 e^–^ onto the central oxo bridge, thus rendering it similar to a free radical O^•–^ (note that the transferred spin density in all other cases does not exceed 0.15 e^–^). This obviously gives rise to a very strong FM superexchange, as expected, and qualitatively resembles an impact of organic free-radical ligands. Quantitatively, the effect is dramatic, however, as the absolute value of *J*_MM_ increases nearly 17 times in this case.

**Figure 3:**
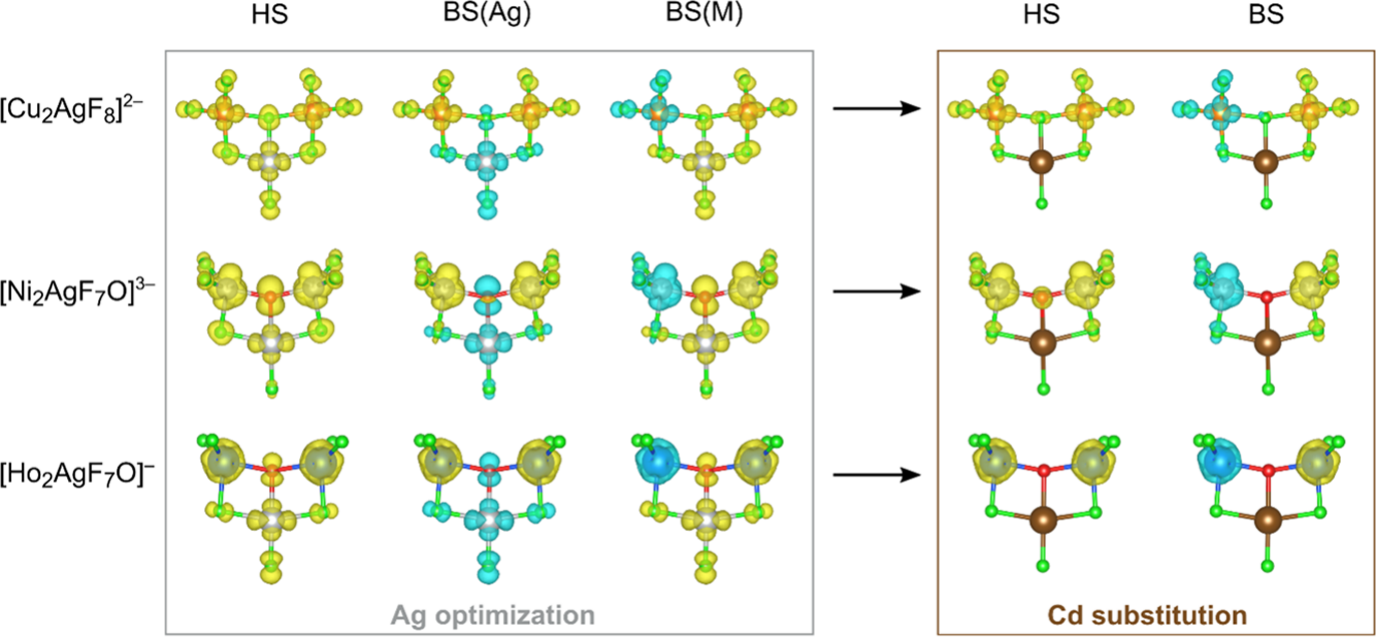
Spin densities for each spin state for three cluster geometries. Top row: Cu(II) with a F^–^ bridge, center row: Ni(II) with an O^2–^ bridge, bottom row: Ho(III) with an O^2–^ bridge. The left panel shows spin densities for the optimized molecules (i.e., with Ag(II) cations), while the after-effect of Ag(II) → Cd(II) substitution is shown in the right panel. Note the dramatic changes in the spin density of all bridging ligands associated with the presence of Ag(II) as compared to those with Cd(II) that greatly affect the M–M superexchange. The spin density isosurface value is set to 0.007 e/Å^3^.

Overall, the impact of Ag(II) on the M–M superexchange is found to be moderate (up to 2.5-fold increase), and it enhances the AFM character of *J*_MM_ in nearly all cases. Aside from influencing the M–M superexchange, the presence of Ag introduces substantial Ag–M superexchange, which may be very strong and, thus, dictates the magnetic GS of the corresponding molecule even if spin frustration is present. However, in the case of the Ni_2_ oxo complex, the effects are much larger and qualitatively opposite (FM instead of AFM superexchange). Comparison to the analogous M_2_-Cd cases suggests that the key reason for the difference is in the fact that Ag(II) drastically polarizes the bridging ligands and introduces spin to the p orbitals, which are involved in the superexchange (Figure 3).

To get further insight into the importance of diverse superexchange pathways, we have looked into modified clusters with bridging ligands selectively removed.

**Figure 4:**
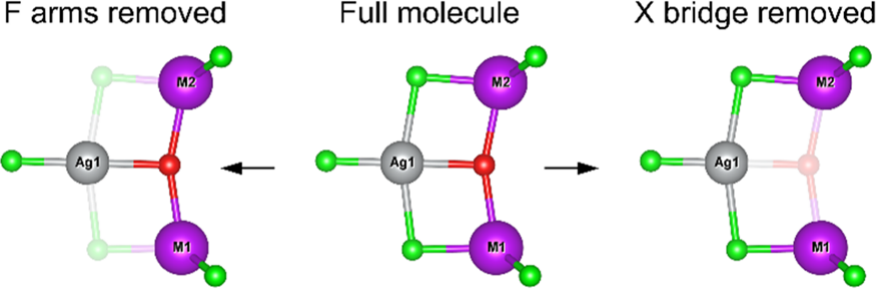
Scheme showing the abstraction of bridging ligands: two outer F atoms (left) or the central X atom (right). M stands for either 3d TM or Ln.

Two main paths for M–M superexchange interaction exist in the model clusters: a short one through a central X ligand and a longer one via two side bridges and the Ag(II) cation. In order to clarify whether any of those is more important, we performed single-point calculations for a geometry corresponding to a previously optimized “full” cluster with either (i) the X ligand removed from the M–X–M path or (ii) the two bridging fluorides eliminated from the M–F–Ag–F–M path. The abstraction is illustrated in Figure 4 and the results are presented in Table 3 (for geometry analysis cf. Table S2 in Supporting Information).

**Table 3:** Calculated Exchange Constants for Full (Optimized) Molecules and after Abstraction of Either the Central X Bridge or the Two Outer F Bridges from F–Ag–F Pathways, in Line with Figure 4^a^

	[M_2_AgF_7_X]^*n*−^ (full molecule)			[M_2_AgF_7_]^*n*−^ (X bridge removed)			[M_2_AgF_5_X]^*n*−^ (outer F removed)		
system	*J*_AgM_	*J*_MM_(Ag)	*J*_MM_(Cd)	*J*_AgM_	*J*_MM_(Ag)	*J*_MM_(Cd)	*J*_AgM_	*J*_MM_(Ag)	*J*_MM_(Cd)
Cu(F)	–285.5	–127.6	–84.7	–602.3	–29.4	4.1	428.3	–236.6	–8.1
Gd(O)	–12.54	–0.146	–0.119	3.16	0.018	0.015	–52.89	–0.161	–0.226
Ni(O)	409.1	1670.6	–100.5	315.3	–42.7	–26.5	86.8	28.0	21.5

a Data shows exchange constants provided in cm^–1^ for M–M interaction before and after Ag → Cd-substitution.

Let us focus on a Cu system with X = F. Here, for an initial cluster we have computed a ca. 51% increase (from *ca.* −85 to *ca.* −128 cm^–1^) in the AFM Cu–Cu interaction upon the Cd(II) → Ag(II) substitution. It turns out that both types of ligand abstraction change the superexchange considerably: (i) in the absence of the central bridge, the M–M superexchange is also AFM but much weaker than for the initial system (ca. −29 cm^–1^) since it is transferred via a longer pathway, while (ii) in the absence of the two outer fluoride ligands, the M–M superexchange is much stronger than for the initial cluster (ca. −237 cm^–1^). Simultaneously, the corresponding Ag–M interactions are even more affected since for (i) it more than doubles from *ca.* −286 to −602 cm^–1^, while for (ii) it becomes strongly FM (ca. +428 cm^–1^) as it now propagates via a bridge with a bond angle of nearly 90°. In any case, the effects of bridge removal are not additive (i.e., the complete system cannot be understood as a simple superposition of components lacking each type of bridging ligand at a time), and they certainly influence each other. The same conclusion holds for the other two clusters scrutinized (M = Gd with X = O and M = Ni with X = O, Table 3). Thus, even for quite simple geometry and symmetry of the clusters, the complexity of their magnetic characteristics is substantial.

In view of noticeable or large effects seen in magnetic properties for Ag(II) systems, we pondered whether the Cu(II) cation could do the same job as Ag(II). This is of particular interest for Ln clusters, as well as for metal oxo clusters (as copper shows strong affinity to oxygen, as silver does to fluorine). Since dozens of structures of Cu(II)-Ln complexes have been described in the literature, we have also explored clusters containing Cu(II) for comparison. The cluster geometry after Ag(II) → Cu(II) substitution generally does not change much, and the superexchange coupling constants in the copper clusters could be calculated (Table S5 in the Supporting Information). It turns out that the *J*_AgM_ values are usually 2 to 3 times larger than the corresponding *J*_CuM_ ones. For example, Gd(III) oxide-bridged complex with Cu(II) exhibits *J*_AgM_ of −3.64 cm^–1^, while the Ag(II) shows *J*_AgM_ of −12.54 cm^–1^. Thus, the integration of Ag(II), rather than Cu(II), into Ln clusters is more appealing in terms of the achieved increase in the strength of magnetic interactions.

As this work is an attempt to motivate the use of Ag(II), which can be seen as an inorganic analogue of an organic free radical, as a spin-polarizer, similar polynuclear spin clusters systems incorporating organic radical bridges obviously constitute important reference systems. A number of Ln clusters with radical ligands have been described in the literature over the past decade. One of the most astounding ones, described by Rinehart et al.,^70^ holds a N_2_^•3–^ ligand bridging two Gd^3+^ centers, which are both coupled antiferromagnetically with the radical with a large exchange constant of −27 cm^–1^. This value can be directly compared to our obtained *J*_AgM_ values, which for the gadolinium cluster yields −12.5 cm^–1^ with X = O^2–^, which is of the same order of magnitude (whereas there is a chemical bond between Gd and N in the published system, in our system Ag(II) is separated from Ln by nonmetal bridges). Unfortunately, the impact of the unusual free-radical bridge, N_2_^•3–^, on the effective intercationic superexchange constant, *J*_MM_, was not determined by the authors.^70^ Yet another impressive attempt to maximize the radical-Ln exchange, ultimately resulted in a complex hosting AFM coupling of −430 cm^–1^ between Yb^3+^ and a bipy^•–^ ligand (2*J* = −920 cm^–1^).^71^ Unfortunately, this system contained only a single Ln center, and so *J*_MM_ is not relevant.

To compare the impact of Ag(II) and organic free radicals, we have run several calculations for systems containing a classical stable organic radical, TEMPO. The additional results presented in Table S6 of the Supporting Information show that the preferred Ln system is the Gd_2_F_4_O cluster with TEMPO attached, where the O(2p) hole lies parallel to the Gd–O–Gd–O(TEMPO) plane. The TEMPO–Gd^3+^ coupling can reach about 11.5 cm^–1^, which is a comparable strength to Ag^2+^–Gd^3+^ coupling, although of an opposite, that is, FM character. On the other hand, the [Ni_2_F_5_·TEMPO]^−^ system shows remarkably strong TEMPO–Ni FM coupling of +229 cm^–1^, which is only about a half of the Ag(II)–Ni(II) coupling of 409 cm^–1^. These results emphasize not only the powerful influence of Ag^2+^ on the coupling between other cationic centers (i.e., via *J*_MM_) but also for coupling itself as a d^9^ radical to other TMs (i.e., via *J*_AgM_) and despite the fact that additional bridges separate Ag and the heterometal centers.

## Conclusions

Our calculations for small hypothetical coordination clusters containing two metal spin centers (M = TM or Ln) bridged by fluoride, oxo, or chloro ligands reveal that the presence of a divalent silver cation can substantially alter the cluster’s magnetic characteristics. Typically, Ag(II) induces a strong polarization to any ligand attached. If tightly connected to the bridging ligand that mediates the M–M superexchange, as is the case in our model clusters, Ag(II) may enhance the M–M superexchange resulting in exchange constants that increase in absolute terms up to 0.2 cm^–1^ for Lns (i.e., often by a factor of 2) and even more than 200 cm^–1^ for 3d TMs. In the extreme case of a Ni_2_ complex with a central oxo bridge, the spin-polarizing effect is so large that it renders the oxo bridge similar to a radical anion and can even change the nature of the interaction (from AFM to FM). Simultaneously, the Ag(II)–M superexchange is typically very strong and occasionally leads to spin frustration in some of the studied clusters. For example, the Ag(II)–Ln superexchange may be comparable to or even surpass the Cu(II)-Ln exchange reported in the literature, reaching *J* values as high as −31.4 cm^–1^ and efficiently increasing the gaps between energy levels of the Ln elements. These results indicate a potential path toward more complex polynuclear clusters with exchange-coupled spin centers that in principle can result in significantly higher multiplet states. Future work will focus on deconvoluting the various coupling mechanisms further and comparing to an extended set of structural analogues. Ag(II) seems to offer an exciting alternative to organic radical ligands in this field.

## Supporting Information Available

Comprehensive computational details, validation of methodology, molecular geometries including superexchange pathways, and Löwdin spin populations and analysis of superexchange interactions, including those for the selected Cu(II) reference systems (PDF)

## Dedication

This work is dedicated to P. Pyykkö on his 80th birthday.

## References

[ref1] Wolf S. A.; Awschalom D. D.; Buhrman R. A.; Daughton J. M.; von Molnár S.; Roukes M. L.; Chtchelkanova A. Y.; Treger D. M. Spintronics: A Spin-Based Electronics Vision for the Future. Science 2001, 294, 1488–1495. 10.1126/science.1065389. 11711666

[ref2] Žutić I.; Fabian J.; Das Sarma S. Spintronics: Fundamentals and Applications. Rev. Mod. Phys. 2004, 76, 323–410. 10.1103/RevModPhys.76.323.

[ref3] Awschalom D. D.; Flatté M. E. Challenges for Semiconductor Spintronics. Nat. Phys. 2007, 3, 153–159. 10.1038/nphys551.

[ref4] Baltz V.; Manchon A.; Tsoi M.; Moriyama T.; Ono T.; Tserkovnyak Y. Antiferromagnetic Spintronics. Rev. Mod. Phys. 2018, 90, 015005 10.1103/RevModPhys.90.015005.

[ref5] Bhatti S.; Sbiaa R.; Hirohata A.; Ohno H.; Fukami S.; Piramanayagam S. N. Spintronics Based Random Access Memory: A Review. Mater. Today 2017, 20, 530–548. 10.1016/j.mattod.2017.07.007.

[ref6] Gomonay E. V.; Loktev V. M. Spintronics of antiferromagnetic systems (Review Article). Low Temp. Phys. 2014, 40, 17–35. 10.1063/1.4862467.

[ref7] Dietl T.; Ohno H. Dilute Ferromagnetic Semiconductors: Physics and Spintronic Structures. Rev. Mod. Phys. 2014, 86, 187–251. 10.1103/RevModPhys.86.187.

[ref8] Bogani L.; Wernsdorfer W. Molecular Spintronics Using Single-Molecule Magnets. Nat. Mater. 2008, 7, 179–186. 10.1038/nmat2133. 18297126

[ref9] Rocha A. R.; García-suárez V. M.; Bailey S. W.; Lambert C. J.; Ferrer J.; Sanvito S. Towards Molecular Spintronics. Nat. Mater. 2005, 4, 335–339. 10.1038/nmat1349. 15750597

[ref10] Sanvito S. Molecular Spintronics. Chem. Soc. Rev. 2011, 40, 3336–3355. 10.1039/C1CS15047B. 21552606

[ref11] Felser C.; Fecher G. H.; Balke B. Spintronics: A Challenge for Materials Science and Solid-State Chemistry. Angew. Chem., Int. Ed. 2007, 46, 668–699. 10.1002/anie.200601815. 17219604

[ref12] Sanvito S.; Rocha A. R. Molecular-Spintronics: The Art of Driving Spin Through Molecules. J. Comput. Theor. Nanosci. 2006, 3, 624–642. 10.1166/jctn.2006.3047.

[ref13] Mallah T.; Thiébaut S.; Verdaguer M.; Veillet P. High- T c Molecular-Based Magnets: Ferrimagnetic Mixed-Valence Chromium(III)-Chromium(II) Cyanides with T c at 240 and 190 Kelvin. Science 1993, 262, 1554–1557. 10.1126/science.262.5139.1554. 17829385

[ref14] Lanthanides and Actinides in Molecular Magnetism; Layfield R. A., Murugesu M., Eds.; John Wiley & Sons: Weinheim, 2015.

[ref15] Clemente-Juan J. M.; Coronado E.; Gaita-Ariño A. Magnetic Polyoxometalates: From Molecular Magnetism to Molecular Spintronics and Quantum Computing. Chem. Soc. Rev. 2012, 41, 7464–7478. 10.1039/C2CS35205B. 22948854

[ref16] Camarero J.; Coronado E. Molecular vs. Inorganic Spintronics: The Role of Molecular Materials and Single Molecules. J. Mater. Chem. 2009, 19, 1678–1684. 10.1039/B819594N.

[ref17] Heersche H. B.; de Groot Z.; Folk J. A.; van der Zant H. S. J.; Romeike C.; Wegewijs M. R.; Zobbi L.; Barreca D.; Tondello E.; Cornia A. Electron Transport through Single Mn_12_ Molecular Magnets. Phys. Rev. Lett. 2006, 96, 206801 10.1103/PhysRevLett.96.206801. 16803192

[ref18] Ishikawa N.; Sugita M.; Ishikawa T.; Koshihara S.; Kaizu Y. Mononuclear Lanthanide Complexes with a Long Magnetization Relaxation Time at High Temperatures: A New Category of Magnets at the Single-Molecular Level. J. Phys. Chem. B 2004, 108, 11265–11271. 10.1021/jp0376065.

[ref19] Murugesu M.; Habrych M.; Wernsdorfer W.; Abboud K. A.; Christou G. Single-Molecule Magnets: A Mn25 Complex with a Record S = 51/2 Spin for a Molecular Species. J. Am. Chem. Soc. 2004, 126, 4766–4767. 10.1021/ja0316824. 15080666

[ref20] Peng J.-B.; Zhang Q.-C.; Kong X.-J.; Zheng Y.-Z.; Ren Y.-P.; Long L.-S.; Huang R.-B.; Zheng L.-S.; Zheng Z. High-Nuclearity 3d-4f Clusters as Enhanced Magnetic Coolers and Molecular Magnets. J. Am. Chem. Soc. 2012, 134, 3314–3317. 10.1021/ja209752z. 22313167

[ref21] McAdams S. G.; Ariciu A.-M.; Kostopoulos A. K.; Walsh J. P. S.; Tuna F. Molecular Single-Ion Magnets Based on Lanthanides and Actinides: Design Considerations and New Advances in the Context of Quantum Technologies. Coord. Chem. Rev. 2017, 346, 216–239. 10.1016/j.ccr.2017.03.015.

[ref22] Frost J. M.; Harriman K. L. M.; Murugesu M. The Rise of 3-d Single-Ion Magnets in Molecular Magnetism: Towards Materials from Molecules?. Chem. Sci. 2016, 7, 2470–2491. 10.1039/C5SC03224E. 28660017 PMC5477015

[ref23] Kowalski N.; Dash S. S.; Sémon P.; Sénéchal D.; Tremblay A.-M. Oxygen Hole Content, Charge-Transfer Gap, Covalency, and Cuprate Superconductivity. Proc. Natl. Acad. Sci. U.S.A. 2021, 118, e2106476118 10.1073/pnas.2106476118. 34593641 PMC8501840

[ref24] Wang L.; He G.; Yang Z.; Garcia-Fernandez M.; Nag A.; Zhou K.; Minola M.; Tacon M. L.; Keimer B.; Peng Y.; Li Y. Paramagnons and High-Temperature Superconductivity in a Model Family of Cuprates. Nat. Commun. 2022, 13, 3163 10.1038/s41467-022-30918-z. 35672416 PMC9174205

[ref25] Grochala W.; Hoffmann R. Real and Hypothetical Intermediate-Valence AgII/AgIII and Ag^II^/Ag^I^ Fluoride Systems as Potential Superconductors. Angew. Chem., Int. Ed. 2001, 40, 2742–2781. 10.1002/1521-3773(20010803)40:15<2742::AID-ANIE2742>3.0.CO;2-X. 29711991

[ref26] Grochala W.; Egdell R. G.; Edwards P. P.; Mazej Z.; Žemva B. On the Covalency of Silver-Fluorine Bonds in Compounds of Silver(I), Silver(II) and Silver(III). ChemPhysChem 2003, 4, 997–1001. 10.1002/cphc.200300777. 14562447

[ref27] Bachar N.; Koteras K.; Gawraczynski J.; Trzciński W.; Paszula J.; Piombo R.; Barone P.; Mazej Z.; Ghiringhelli G.; Nag A.; Zhou K.-J.; Lorenzana J.; van der Marel D.; Grochala W. Charge-Transfer and dd excitations in AgF_2_. Phys. Rev. Res. 2022, 4, 023108 10.1103/PhysRevResearch.4.023108.

[ref28] Yan F.; Chen Z. Magnetic Coupling Constants and Spin Density Maps for Heterobinuclear Complexes GdCu(OTf)_3_(Bdmap)_2_(H_2_O)·THF, [Gd(C_4_H_7_ON)_4_(H_2_O)_3_][Fe(CN)_6_]·2H_2_O, and [Gd(C_4_H_7_ON)_4_(H_2_O)_3_][Cr(CN)_6_]·2H_2_O: A Density Functional Study. J. Phys. Chem. A 2000, 104, 6295–6300. 10.1021/jp994093m.

[ref29] Rajaraman G.; Totti F.; Bencini A.; Caneschi A.; Sessoli R.; Gatteschi D. Density functional studies on the exchange interaction of a dinuclear Gd(iii)-Cu(ii) complex: method assessment, magnetic coupling mechanism and magneto-structural correlations. Dalton Trans. 2009, 0, 3153–3161. 10.1039/B817540C. 19421617

[ref30] Cirera J.; Ruiz E. Exchange Coupling in Cu^II^Gd^III^ Dinuclear Complexes: A Theoretical Perspective. Comptes Rendus Chim 2008, 11, 1227–1234. 10.1016/j.crci.2008.04.012.

[ref31] Long J.; Habib F.; Lin P.-H.; Korobkov I.; Enright G.; Ungur L.; Wernsdorfer W.; Chibotaru L. F.; Murugesu M. Single-Molecule Magnet Behavior for an Antiferromagnetically Superexchange-Coupled Dinuclear Dysprosium(III) Complex. J. Am. Chem. Soc. 2011, 133, 5319–5328. 10.1021/ja109706y. 21425794

[ref32] Reger D. L.; Pascui A. E.; Smith M. D.; Jezierska J.; Ozarowski A. Dinuclear Complexes Containing Linear M-F-M [M = Mn(II), Fe(II), Co(II), Ni(II), Cu(II), Zn(II), Cd(II)] Bridges: Trends in Structures, Antiferromagnetic Superexchange Interactions, and Spectroscopic Properties. Inorg. Chem. 2012, 51, 11820–11836. 10.1021/ic301757g. 23043562

[ref33] Pedersen K. S.; Sigrist M.; Weihe H.; Bond A. D.; Thuesen C. Aa.; Simonsen K. P.; Birk T.; Mutka H.; Barra A.-L.; Bendix J. Magnetic Interactions through Fluoride: Magnetic and Spectroscopic Characterization of Discrete, Linearly Bridged [Mn^III^_2_(μ-F)F_4_(Me_3_tacn)_2_](PF_6_). Inorg. Chem. 2014, 53, 5013–5019. 10.1021/ic500049w. 24601580

[ref34] Pedersen K. S.; Bendix J. Molecular Fluoride-Bridged 3d-4f Complexes and Their Magnetic Properties. In Photonic and Electronic Properties of Fluoride Materials; Tressaud A., Poeppelmeier K., Eds.; Elsevier: Boston, 2016; pp 213–230.

[ref35] Pedersen K. S.; Sørensen M. A.; Bendix J. Fluoride-Coordination Chemistry in Molecular and Low-Dimensional Magnetism. Coord. Chem. Rev. 2015, 299, 1–21. 10.1016/j.ccr.2015.03.024.

[ref36] Hayden S. M.; Aeppli G.; Osborn R.; Taylor A. D.; Perring T. G.; Cheong S.-W.; Fisk Z. High-Energy Spin Waves in La_2_CuO_4_. Phys. Rev. Lett. 1991, 67, 3622–3625. 10.1103/PhysRevLett.67.3622. 10044782

[ref37] Guarise M.; Dalla Piazza B.; Moretti Sala M.; Ghiringhelli G.; Braicovich L.; Berger H.; Hancock J. N.; van der Marel D.; Schmitt T.; Strocov V. N.; Ament L. J. P.; van den Brink J.; Lin P.-H.; Xu P.; Rønnow H. M.; Grioni M. Measurement of Magnetic Excitations in the Two-Dimensional Antiferromagnetic Sr_2_CuO_2_Cl_2_ Insulator Using Resonant X-Ray Scattering: Evidence for Extended Interactions. Phys. Rev. Lett. 2010, 105, 157006 10.1103/PhysRevLett.105.157006. 21230933

[ref38] Okada K.; Kotani A.; Maiti K.; Sarma D. D. Cu 2p Core-Level Photoemission Spectrum of Sr_2_CuO_3_. J. Phys. Soc. Jpn. 1996, 65, 1844–1848. 10.1143/JPSJ.65.1844.

[ref39] Gawraczyński J.; Kurzydłowski D.; Ewings R. A.; Bandaru S.; Gadomski W.; Mazej Z.; Ruani G.; Bergenti I.; Jaroń T.; Ozarowski A.; Hill S.; Leszczyński P. J.; Tokár K.; Derzsi M.; Barone P.; Wohlfeld K.; Lorenzana J.; Grochala W. Silver Route to Cuprate Analogs. Proc. Natl. Acad. Sci. U.S.A. 2019, 116, 1495–1500. 10.1073/pnas.1812857116. 30651308 PMC6358696

[ref40] Grzelak A.; Su H.; Yang X.; Kurzydłowski D.; Lorenzana J.; Grochala W. Epitaxial Engineering of Flat Silver Fluoride Cuprate Analogs. Phys. Rev. Mater. 2020, 4, 084405 10.1103/PhysRevMaterials.4.084405.

[ref41] Kurzydłowski D.; Mazej Z.; Jagličić Z.; Filinchuk Y.; Grochala W. Structural Transition and Unusually Strong Antiferromagnetic Superexchange Coupling in Perovskite KAgF_3_. Chem. Commun. 2013, 49, 6262–6264. 10.1039/C3CC41521J. 23598949

[ref42] Kurzydłowski D.; Grochala W. Prediction of Extremely Strong Antiferromagnetic Superexchange in Silver(II) Fluorides: Challenging the Oxocuprates(II). Angew. Chem. 2017, 129, 10248–10251. 10.1002/ange.201700932. 28485841

[ref43] Kurzydłowski D.; Grochala W. Large Exchange Anisotropy in Quasi-One-Dimensional Spin-1/2 Fluoride Antiferromagnets with a dz^2^ Ground State. Phys. Rev. B 2017, 96, 155140 10.1103/PhysRevB.96.155140.

[ref44] Kurzydłowski D.; Derzsi M.; Barone P.; Grzelak A.; Struzhkin V.; Lorenzana J.; Grochala W. Dramatic enhancement of spin-spin coupling and quenching of magnetic dimensionality in compressed silver difluoride. Chem. Commun. 2018, 54, 10252–10255. 10.1039/C8CC05002C. 30039135

[ref45] Koteras K.; Gawraczyński J.; Tavčar G.; Mazej Z.; Grochala W. Crystal Structure, Lattice Dynamics and Superexchange in MAgF_3_ 1D Antiferromagnets (M = K, Rb, Cs) and a Rb_3_Ag_2_F_7_ Ruddlesden–Popper Phase. CrystEngComm 2022, 24, 1068–1077. 10.1039/D1CE01545A.

[ref46] Jaroń T.; Grochala W. Prediction of Giant Antiferromagnetic Coupling in Exotic Fluorides of Ag^II^. Phys. Status Solidi RRL 2008, 2, 71–73. 10.1002/pssr.200701286.

[ref47] Demir S.; Jeon I.-R.; Long J. R.; Harris T. D. Radical Ligand-Containing Single-Molecule Magnets. Coord. Chem. Rev. 2015, 289–290, 149–176. 10.1016/j.ccr.2014.10.012.

[ref48] Caneschi A.; Gatteschi D.; Sessoli R.; Rey P. Toward Molecular Magnets: The Metal-Radical Approach. Acc. Chem. Res. 1989, 22, 392–398. 10.1021/ar00167a004.

[ref49] Herebian D.; Wieghardt K. E.; Neese F. Analysis and Interpretation of Metal-Radical Coupling in a Series of Square Planar Nickel Complexes: Correlated Ab Initio and Density Functional Investigation of [Ni(LISQ)_2_] (LISQ=3,5-Di-Tert-Butyl-o-Diiminobenzosemiquinonate(1-)). J. Am. Chem. Soc. 2003, 125, 10997–11005. 10.1021/ja030124m. 12952481

[ref50] Gendron F.; Autschbach J.; Malrieu J.-P.; Bolvin H. Magnetic Coupling in the Ce(III) Dimer Ce_2_(COT)_3_. Inorg. Chem. 2019, 58, 581–593. 10.1021/acs.inorgchem.8b02771. 30565926

[ref51] Jang S.-H.; Sano R.; Kato Y.; Motome Y. Computational Design of f-Electron Kitaev Magnets: Honeycomb and Hyperhoneycomb Compounds A_2_PrO_3_ (A=Alkali Metals). Phys. Rev. Mater. 2020, 4, 104420 10.1103/PhysRevMaterials.4.104420.

[ref52] Peters L.; Ghosh S.; Sanyal B.; van Dijk C.; Bowlan J.; de Heer W.; Delin A.; Di Marco I.; Eriksson O.; Katsnelson M. I.; Johansson B.; Kirilyuk A. Magnetism and Exchange Interaction of Small Rare-Earth Clusters; Tb as a Representative. Sci. Rep. 2016, 6, 19676 10.1038/srep19676. 26795239 PMC4726341

[ref53] Kahn M. L.; Ballou R.; Porcher P.; Kahn O.; Sutter J.-P. Analytical Determination of the Ln–Aminoxyl Radical Exchange Interaction Taking into Account Both the Ligand-Field Effect and the Spin–Orbit Coupling of the Lanthanide Ion (Ln=Dy^III^ and Ho^III^). Chem.—Eur. J. 2002, 8, 525–531. 10.1002/1521-3765(20020118)8:2<525::AID-CHEM525>3.0.CO;2-L. 11843164

[ref54] Benelli C.; Gatteschi D. Magnetism of Lanthanides in Molecular Materials with Transition-Metal Ions and Organic Radicals. Chem. Rev. 2002, 102, 2369–2388. 10.1021/cr010303r. 12059272

[ref55] Nakamura T.; Kanetomo T.; Ishida T. Strong Antiferromagnetic Interaction in a Gadolinium(III) Complex with Methoxy-TEMPO Radical: A Relation between the Coupling and the Gd-O-N Angle. Inorg. Chem. 2021, 60, 535–539. 10.1021/acs.inorgchem.0c02568. 33382248

[ref56] Lutsenko I. A.; Kiskin M. A.; Nikolaevskii S. A.; Starikova A. A.; Efimov N. N.; Khoroshilov A. V.; Bogomyakov A. S.; Ananyev I. V.; Voronina J. K.; Goloveshkin A. S.; Sidorov A. A.; Eremenko I. L. Ferromagnetically Coupled Molecular Complexes with a Co^II^_2_Gd^III^ Pivalate Core: Synthesis, Structure, Magnetic Properties and Thermal Stability. ChemistrySelect 2019, 4, 14261–14270. 10.1002/slct.201904585.

[ref57] Singh M. K.; Rajeshkumar T.; Kumar R.; Singh S. K.; Rajaraman G. Role of (1,3) {Cu-Cu} Interaction on the Magneto-Caloric Effect of Trinuclear {Cu^II^-Gd^III^-Cu^II^} Complexes: Combined DFT and Experimental Studies. Inorg. Chem. 2018, 57, 1846–1858. 10.1021/acs.inorgchem.7b02775. 29388766

[ref58] Liu D.-P.; Lin X.-P.; Zhang H.; Zheng X.-Y.; Zhuang G.-L.; Kong X.-J.; Long L.-S.; Zheng L.-S. Magnetic Properties of a Single-Molecule Lanthanide-Transition-Metal Compound Containing 52 Gadolinium and 56 Nickel Atoms. Angew. Chem. 2016, 128, 4608–4612. 10.1002/ange.201601199. 26923173

[ref59] Sweet L. E.; Roy L. E.; Meng F.; Hughbanks T. Ferromagnetic Coupling in Hexanuclear Gadolinium Clusters. J. Am. Chem. Soc. 2006, 128, 10193–10201. 10.1021/ja0617690. 16881649

[ref60] Yazyev O. V.; Helm L.; Malkin V. G.; Malkina O. L. Quantum Chemical Investigation of Hyperfine Coupling Constants on First Coordination Sphere Water Molecule of Gadolinium(III) Aqua Complexes. J. Phys. Chem. A 2005, 109, 10997–11005. 10.1021/jp053825+. 16331943

[ref61] Modak R.; Sikdar Y.; Bieńko A.; Witwicki M.; Jerzykiewicz M.; Goswami S. Family of Mn^III^_4_Ln^III^_2_ (Ln^III^=Sm^III^, Gd^III^, Dy^III^) Coordination Clusters: Experimental and Theoretical Investigations. Polyhedron 2016, 119, 202–215. 10.1016/j.poly.2016.08.050.

[ref62] Pantazis D. A.; Neese F. All-Electron Scalar Relativistic Basis Sets for the Lanthanides. J. Chem. Theory Comput. 2009, 5, 2229–2238. 10.1021/ct900090f. 26616609

[ref63] Becke A. D. Density-functional thermochemistry. III. The role of exact exchange. J. Chem. Phys. 1993, 98, 5648–5652. 10.1063/1.464913.

[ref64] Lee C.; Yang W.; Parr R. G. Development of the Colle-Salvetti Correlation-Energy Formula into a Functional of the Electron Density. Phys. Rev. B: Condens. Matter Mater. Phys. 1988, 37, 785–789. 10.1103/PhysRevB.37.785. 9944570

[ref65] Rolfes J. D.; Neese F.; Pantazis D. A. All-electronscalar relativistic basis sets for the elements Rb-Xe. J. Comput. Chem. 2020, 41, 1842–1849. 10.1002/jcc.26355. 32484577

[ref66] Noodleman L. Valence Bond Description of Antiferromagnetic Coupling in Transition Metal Dimers. J. Chem. Phys. 1981, 74, 5737–5743. 10.1063/1.440939.

[ref67] Skinner H. B.; Searcy A. W. Demonstration of the Existence of La_2_F_6_ Gas and Determination of Its Stability. J. Phys. Chem. 1971, 75, 108–111. 10.1021/j100671a019.

[ref68] Akdeniz Z.; Gaune-Escard M.; Tosi M. P. Static and Dynamic Structure of Molecular Monomers and Dimers of the Rare-Earth Fluorides. Z. Naturforsch., A: Phys. Sci. 2001, 56, 381–385. 10.1515/zna-2001-0507.

[ref69] Solomonik V. G.; Smirnov A. N. Structure and Energy Stability of Lanthanum and Lutetium Trihalide Dimer Molecules. J. Struct. Chem. 2005, 46, 973–978. 10.1007/s10947-006-0230-y.

[ref70] Rinehart J. D.; Fang M.; Evans W. J.; Long J. R. Strong Exchange and Magnetic Blocking in N_2_^3–^-Radical-Bridged Lanthanide Complexes. Nat. Chem. 2011, 3, 538–542. 10.1038/nchem.1063. 21697874

[ref71] Lukens W. W.; Magnani N.; Booth C. H. Application of the Hubbard Model to Cp*_2_Yb(Bipy), a Model System for Strong Exchange Coupling in Lanthanide Systems. Inorg. Chem. 2012, 51, 10105–10110. 10.1021/ic300037q. 22988887

