# Supplementary material for: Ag(II) as Spin Super-Polarizer in Molecular Spin Clusters
Source: J Phys Chem A. 2022 Dec 15;126(51):9618–26. doi: 10.1021/acs.jpca.2c06032 (PMC9806831; doi:10.1021/acs.jpca.2c06032)
Supplement: Supplementary file 1 — jp2c06032_si_001.pdf [file jp2c06032_si_001.pdf]

Ag(II) as Spin Super-Polarizer in Molecular Spin Clusters

Mateusz Domański[a], Jan van Leusen[b], Marvin Metzelaars,[b] Paul Kögerler*[b], and Wojciech Grochala*[a]

[a] Prof. Wojciech Grochala, Mateusz Domański
Center of New Technologies
University of Warsaw
Zwirki i Wigury 93, 02089 Warsaw Poland
E-mail: w.grochala@cent.uw.edu.pl

[b] Dr. Marvin Matzelaars, Dr. Jan van Leusen, Prof. Paul Kögerler
Institut für Anorganische Chemie
RWTH Aachen University,
52056 Aachen, Germany
E-mail: paul.koegerler@ac.rwth-aachen.de

1. Methods

The largest effort in theoretical research on magnetic clusters containing lanthanides is spent for a correct description of 4f electrons. These levels are subject to a splitting originating from ligand (crystal) field and spin-orbit interactions.[1] Recently there have been published many computational studies on spin-spin coupling in molecular systems containing one or more lanthanide cations, i.a. : cerium[2], praseodymium[3], europium[4], gadolinium[4], terbium[4,5], dysprosium[4,6], holmium[4,6]. In all of these studies[2–6] the spin-orbit coupling effects have been considered. However, for the most gadolinium studies[7–14] where the spin-orbit coupling effect is predicted to be weak, the scalar-relativistic methods and the Ising type Hamiltonian were used. The issue with spin-orbit coupling is that it can be relatively easy employed for single centre systems, and then usually CAS is nowadays used for this. However, on larger systems this approach becomes often too expensive, but still there is a necessity to correctly account for relativistic effects[9]. Due to extensive range of hypothetical molecules considered in this study, we chose an approach of robust hybrid DFT approach together with a scalar relativistic Hamiltonian and all-electron basis set.

The study is based on density functional theory (DFT) calculations using ORCA 4.2.1. program[15] applying the frequently used B3LYP functional[16,17]. Calculations were performed with a "very tight" SCF convergence procedure and with the most accurate numerical integration grid available (Lebedev 770 points). The relativistic effects were included with the use of 0th order regular approximation (ZORA) Hamiltonian[18,19] with one-centre approximation as implemented in ORCA. The ZORA Hamiltonian including spin-orbit effects is only available for closed-shell molecules in DFT calculations, thus we applied scalar relativistic ZORA Hamiltonian. Segmented all-electron relativistically contracted basis set SARC-ZORA-TZVP was used for Ag and lanthanides and relativistically recontracted ZORA-def2-TZVP basis for the lighter elements[20,21]. Moreover, properties calculated with the SARC-ZORA-TZVP compact basis set are consistent with experimentally measured properties for heavy elements and their molecules, *i.a.* ionization potentials, despite the calculations were performed with scalar relativistic Hamiltonian ZORA[20,21]. Especially, in contrast to heavier elements, the effects of spin-orbit coupling in the lanthanides are not usually considered of critical importance because they are smaller in magnitude than the correlation effects arising from the different electronic configurations.[21–24] Moreover, for the methods we use here (hybrid DFT B3LYP), it is not expected that inclusion of spin-orbit corrections would lead to systematic improvement of the computed values. [21–24] Thus, the spin-orbit coupling was neglected to focus on the impact of Ag^{2+} on the 4f cations interactions.

To calculate the strength of spin-spin interactions the Ising model Hamiltonian was used in the form $H = -J_{\langle ij \rangle} \sum_{i,j} S_{z,i} S_{z,j}$ (with $J_{\langle ij \rangle}$=$J_{\langle ji \rangle}$), where $J_{\langle ij \rangle}$ is antiferromagnetic coupling constant between the closest neighbouring magnetic centres i and j when $J_{ij} < 0$. The exchange spin coupling parameters J were calculated using the "broken symmetry" (BS) formalism[25] by calculation of all the possible spin states in each system and solving the set of linear equations. Due to the fact that only a part of molecular geometries were perfectly symmetric (**Table S2**), the exchange constants between silver and two other cations in principle are not equal. Because of that, in the model molecules of AgM_2F_7X (X = F, Cl, O) three types of exchange constants were applied, $J_{Ag\text{-}M1}$, $J_{Ag\text{-}M2}$ and $J_{M\text{-}M}$, these results are presented in **Table S3**.

For each chemical system, the geometry optimization for each possible spin-state was performed in a search for the ionic and electronic ground state simultaneously. To apply broken symmetry formalism, the energies of excited spin states were calculated in a ground state molecular geometry. For every system, possible ground states are; high spin HS, silver broken-symmetry state BS(Ag) (with Ag having opposite spin) and the other metal broken-symmetry state BS(M) (with M1 or M2 having opposite spin). Importantly, for each spin state the energy was thoroughly optimized to the lowest value, in particular by ensuring that all of the orbitals are occupied in the same manner. This consistency further diminishes effect of spin-orbit coupling, as all spin-states would be affected with the same way since all orbitals are similarly occupied both in Ag and in Cd-substituted complexes. To check orbital occupancy, Löwdin spin population analyses[26] were carried out (as implemented in Orca), with f-orbital spin populations carefully inspected during calculations, see **Table S7**, **Table S8**, **Table S9**, **Table S10**, **Table S11** and **Table S12**. Finally, to evaluate the influence of Ag^{2+} spin polarizing properties on the f-electrons, the Ag^{2+} cations were substituted with Cd^{2+} cations. In each substituted system $J_{M\text{-}M}$ was recalculated – changes upon substitution indicate, whether or not, Ag^{2+} influences this interaction (**Table 1** in the main paper). To highlight influence of Ag, we also presented spin population on the key atoms, see **Table S4**.

Finally, we have conducted a series of calculations to validate our methodology. Since similar approaches have been successfully utilized in the past for 3d and 4d spin interactions, we assume that in these cases the methods are robust. Thus, here the focus has been focused especially on Ln-Ln and TM-Ln interactions. We applied the method described above for the molecules studied previously in the literature for which experimental data is known. **Table S1** shows comparison of our results to the reference data. The B3LYP/ZORA calculated parameters show good qualitative and fair semi-quantitative agreement with experimental data, the latter itself often having a considerable margin of error (though we notice that experimental errors are rarely provided). Computations with the chosen method yields results within ~1 cm^{-1} despite the fact, that molecular geometries of the complexes were not optimized here, and periodic character of the crystalline systems was ignored. Since the current study relies on Ag/Cd and Cu/Zn substitutions, substantial error cancelling is expected and the major property trends are clearly detectable using our approach.

Table S1. Comparison of results calculated here (B3LYP/ZORA) to reference data of TM-Ln or Ln-Ln complexes.

Molecule	Basis	J(Cu-Ln)^{calc} / cm^{-1}	J(Cu-Ln)^{ref} / cm^{-1}	J(Ln-Ln)^{calc} / cm^{-1}	J(Ln-Ln)^{ref} / cm^{-1}	Δ / cm^{-1}	Ref.
Gd Cu C_{12} H_{16} N_5 O_{13}	SARC-TZVP(Gd), TZVP(Cu, C, H, N, O)	4.79	3.36 ^{a}	-	-	1.43	[27]
Gd Cu C_{45} H_{65} N_2 O_{11}	SARC-TZVP(Gd), TZVP(Cu,N,O), SVP(C,H)	3.20	4.20 ^{b}	-	-	-1.00	[28]
Gd Cu C_{32} H_{38} F_9 N_2 O_{12}	SARC-TZVP(Gd), TZVP(Cu, C, H, F, N, O)	5.14	4.42 ^{b}	-	-	0.72	[29]
Gd Cu_2 C_{40} H_{32} F_9 N_4 O_{10}	SARC-TZVP(Gd), TZVP(Cu, C, H, F, N, O)	2.75	3.54 ^{b}	-	-	-0.79	[29]
Tb_2 C_{40} H_{46} N_8 O_{14}	SARC-TZVP(Tb), TZVP(N,O), SVP(C, H)	-	-	-0.43	-0.10 ^{c}	-0.33	[4]
Gd_2 C_{40} H_{46} N_8 O_{14}	SARC-TZVP(Gd), TZVP(N,O), SVP(C, H)	-	-	-0.28	-0.08 ^{c}	-0.20	[4]

^{a} experimental value, based on magnetostructural correlation of dihedral angle and *J*, correl. coeff. R^2 = 0.9145.

^{b} experimental value, least-square fitting to magnetization data, no correlation coefficient provided.

^{c} value calculated on CASSCF/DKH level of theory with Lines Hamiltonian including Spin-Orbit coupling, which results fit the experimental data in the reference[4]. No correlation coefficients provided.

2. Additional data

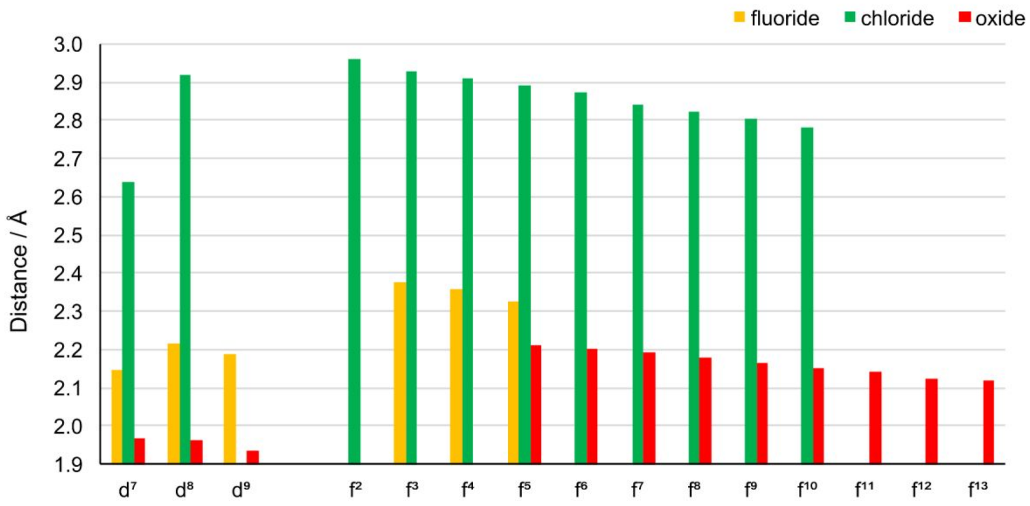

Figure S1. Lengths of Ag-X bonds (X – a bridging ligand) in the optimized clusters studied in this work.

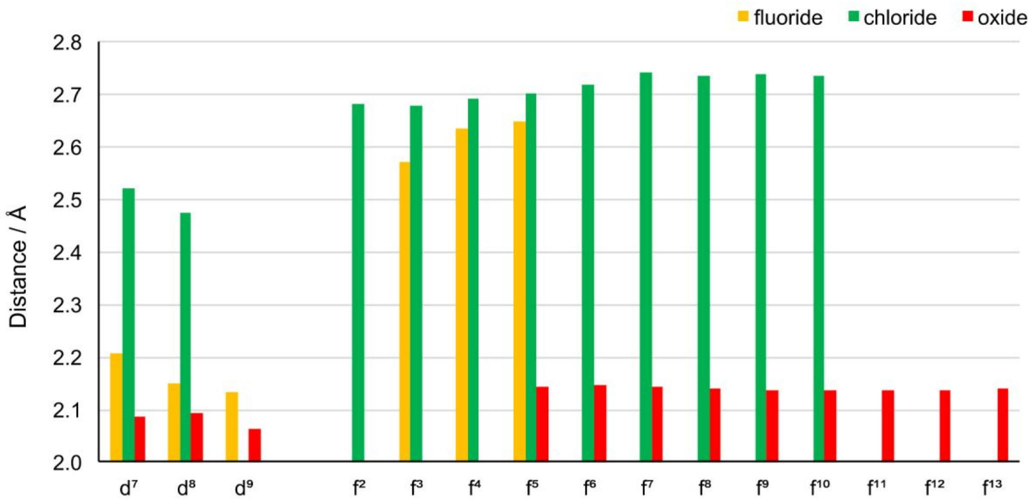

Figure S2. Average length of M-X bonds (X – a bridging ligand) in the optimized clusters studied in this work.

Table S2. The key bond distances (in Å) an angles (in degrees) in the optimized clusters studied in this work (X – a bridging ligand). Difference d(M^1-x)–d(M^2-X) shows the asymmetry of the clusters. Symbols in square brackets indicate the next element in the path.

				Ag-X-M^1, Ag-X-M^2 and M^1-X-M^2 bridges						M^1-F-Ag bridge			M^2-F-Ag bridge					
Ligand	M	v.e.	GS	d(Ag-X)	d(M^1-X)	d(M^2-X)	∠ (M^2-X-M^1)	∠ (M^1-X-Ag)	∠ (M^2-X-Ag)	d(M^1-F) [-Ag]	d(Ag-F) [-M^1]	∠ (M^1-F-Ag)	d(M^2-F) [-Ag]	d(Ag-F) [-M^2]	∠ (M^2-F-Ag)	d(M^1-Ag)	d(M^2-Ag)	Difference d(M^1-X)-d(M^2-X)
	Co	d^7	BS(Ag)	2.206	2.146	2.147	168.6	96.4	94.9	2.032	2.131	102.4	2.014	2.136	101.2	3.246	3.208	-0.001
	Ni	d^8	BS(Ag)	2.149	2.215	2.216	168.4	95.8	95.8	1.984	2.131	103.4	1.985	2.131	103.5	3.238	3.240	-0.001
F^-	Cu	d^9	BS(Ag)	2.132	2.188	2.186	164.2	97.8	98.0	1.987	2.131	104.5	1.986	2.131	104.6	3.257	3.259	0.002
	Nd	f^3	BS(Ln)	2.571	2.378	2.375	168.9	95.4	95.7	2.263	2.143	112.4	2.268	2.143	112.6	3.662	3.671	0.003
	Pm	f^4	BS(Ag)	2.635	2.355	2.356	171.9	94.3	94.3	2.249	2.142	113.1	2.250	2.141	113.1	3.664	3.664	-0.001
	Sm	f^5	HS	2.647	2.325	2.325	171.2	94.4	94.4	2.238	2.144	113.1	2.237	2.143	113.1	3.655	3.655	0.000
	Co	d^7	BS(Ag)*	2.086	1.969	1.969	156.2	101.9	101.9	2.086	2.275	92.4	2.086	2.275	92.4	3.150	3.150	0.000
	Ni	d^8	HS	2.093	1.962	1.962	154.9	102.5	102.5	2.027	2.296	93.9	2.027	2.296	93.9	3.164	3.164	0.000
	Cu	d^9	BS(Ag)*	2.064	1.934	1.934	149.2	105.4	105.4	2.111	2.224	94.3	2.099	2.227	94.7	1.181	3.180	0.000
	Sm	f^5	BS(Ag)	2.142	2.209	2.209	159.4	100.3	100.3	2.210	2.281	96.1	2.210	2.281	96.1	3.341	3.340	0.000
	Eu	f^6	BS(Ag)	2.147	2.201	2.201	160.9	99.6	99.5	2.203	2.284	95.5	2.203	2.283	95.5	3.320	3.320	0.000
O^{2-}	Gd	f^7	BS(Ag)	2.142	2.192	2.192	160.5	99.8	99.8	2.186	2.289	95.5	2.186	2.289	95.6	3.314	3.314	0.000
	Tb	f^8	BS(Ln)	2.140	2.181	2.173	160.2	99.9	99.9	2.164	2.293	95.8	2.175	2.290	95.3	3.308	3.301	0.008
	Dy	f^9	BS(Ln)	2.138	2.166	2.165	160.0	99.9	100.1	2.156	2.290	95.6	2.155	2.290	95.8	3.295	3.299	0.001
	Ho	f^{10}	HS	2.137	2.152	2.152	160.2	99.9	99.9	2.144	2.292	95.4	2.144	2.291	95.4	3.283	3.282	0.000
	Er	f^{11}	BS(Ag)	2.137	2.142	2.142	160.7	99.7	99.7	2.129	2.293	95.3	2.129	2.293	95.3	3.269	3.270	0.000
	Tm	f^{12}	HS	2.136	2.125	2.125	160.7	99.7	99.6	2.115	2.298	95.0	2.114	2.299	95.0	3.256	3.255	0.000
	Yb	f^{13}	HS	2.139	2.118	2.119	161.7	99.2	99.2	2.105	2.301	94.6	2.105	2.301	94.6	3.241	3.241	-0.001
	Co	d^7	BS(M)	2.520	2.643	2.637	163.9	82.2	81.8	1.990	2.147	110.2	1.984	2.150	109.4	3.394	3.376	0.006
	Ni	d^8	BS(Ag)	2.473	2.918	2.917	159.7	79.9	79.9	1.935	2.167	115.9	1.935	2.166	115.8	3.478	3.476	0.001
	Pr	f^2	BS(Ag)	2.683	2.961	2.960	169.1	84.5	84.6	2.243	2.180	118.5	2.244	2.181	118.5	3.802	3.802	0.001
	Nd	f^3	BS(Ln)	2.680	2.929	2.925	169.4	84.6	84.8	2.228	2.178	118.0	2.233	2.179	118.1	3.778	3.785	0.004
	Pm	f^4	BS(Ag)	2.691	2.908	2.908	168.5	84.3	84.3	2.214	2.178	117.7	2.214	2.178	117.7	3.759	3.759	0.000
Cl^-	Sm	f^5	BS(Ln)	2.702	2.902	2.877	168.1	83.8	84.3	2.203	2.176	117.6	2.200	2.180	117.5	3.745	3.744	0.025
	Eu	f^6	HS	2.719	2.873	2.873	167.2	83.6	83.6	2.186	2.176	117.5	2.186	2.176	117.4	3.728	3.728	0.000
	Gd	f^7	HS	2.741	2.840	2.840	167.1	83.6	83.6	2.175	2.174	117.6	2.175	2.174	117.6	3.720	3.720	0.000
	Tb	f^8	HS	2.734	2.830	2.817	167.1	83.4	83.7	2.155	2.178	117.3	2.165	2.178	117.1	3.701	3.705	0.013
	Dy	f^9	HS	2.738	2.804	2.804	167.0	83.5	83.5	2.146	2.178	117.2	2.146	2.178	117.2	3.691	3.691	0.000
	Ho	f^{10}	HS	2.734	2.781	2.781	167.0	83.5	83.5	2.134	2.181	116.7	2.134	2.180	116.7	3.673	3.673	0.000

Table S3. Computed data for each considered system: valence electron configuration (v.e.), ground state configuration (GS), values of silver-metal and metal-metal superexchange constants, (J_{MM} also after Ag(II) → Cd(II) substitution). Silver-metal exchange couplings are here separated to account for asymmetry in molecular geometries.

Ligand	Metal	v.e.	GS	$J_{AgM(1)}$ / cm^{-1}	$J_{AgM(2)}$ / cm^{-1}	$J_{AgM(1)} - J_{AgM(2)}$ / cm^{-1}	J_{MM}(Ag) / cm^{-1}	J_{MM}(Cd) / cm^{-1}
F^-	Co	d^7	BS(Ag)	23.106	58.964	-35.86	-17.252	-18.161
	Ni	d^8	BS(Ag)	-43.247	-45.562	2.31	-12.482	-11.128
	Cu	d^9	BS(Ag)	-278.009	-292.910	14.90	-127.620	-84.671
	Nd	f^3	BS(Ln)	-19.678	0.702	-20.38	-0.112	-0.175
	Pm	f^4	BS(Ag)	-1.752	-1.801	0.05	-0.278	-0.262
	Sm	f^5	HS	2.686	2.696	-0.01	-0.185	-0.194
O^{2-}	Co	d^7	BS(Ag)*	326.472	326.450	0.02	-71.121	-31.086
	Ni	d^8	HS	409.157	408.949	0.21	1670.595	-100.520
	Cu	d^9	BS(Ag)*	-215.527	-294.961	79.43	-608.671	-380.953
	Sm	f^5	BS(Ag)	-30.147	-29.743	-0.40	-1.073	-1.018
	Eu	f^6	BS(Ag)	-22.619	-22.588	-0.03	-0.016	0.084
	Gd	f^7	BS(Ag)	-12.555	-12.531	-0.02	-0.146	-0.119
	Tb	f^8	BS(Ln)	2.465	-11.068	13.53	-0.160	-0.130
	Dy	f^9	BS(Ln)	-1.691	-1.239	-0.45	-0.293	-0.197
	Ho	f^{10}	HS	2.867	2.827	0.04	-0.281	-0.116
	Er	f^{11}	BS(Ag)	-9.050	-8.936	-0.11	-0.289	-0.131
	Tm	f^{12}	HS	21.777	21.633	0.14	-0.893	-0.826
	Yb	f^{13}	HS	19.683	19.537	0.15	0.326	0.540
Cl^-	Co	d^7	BS(M)	3.495	11.856	-8.36	-32.337	-31.521
	Ni	d^8	BS(Ag)	-83.176	-82.998	-0.18	-2.653	-1.136
	Pr	f^2	BS(Ag)	-31.398	-31.477	0.08	-0.395	-0.334
	Nd	f^3	BS(Ln)	-12.481	3.531	-16.01	-0.361	-0.196
	Pm	f^4	BS(Ag)	-1.943	-1.953	0.01	-0.279	-0.275
	Sm	f^5	BS(Ln)	1.530	2.680	-1.15	-0.090	-0.122
	Eu	f^6	HS	2.893	2.923	-0.03	-0.087	-0.080
	Gd	f^7	HS	2.106	2.111	0.00	-0.096	-0.092
	Tb	f^8	HS	2.070	7.780	-5.71	-0.095	-0.090
	Dy	f^9	HS	6.880	6.880	0.00	-0.078	-0.071
	Ho	f^{10}	HS	1.662	1.917	-0.26	-0.044	-0.038

Table S4. Löwdin spin populations projected on the key atoms in [AgM_2F_7X] clusters in a high-spin state. Spin populations for Cd-substituted molecules (only of high-spin state) together with the ratio highlight the impact of Ag^{2+} on bridging atom spin-polarization. In case of cluster asymmetry, the M spin populations were averaged.

Ligand	Metal	v.e.	GS	Ag	M	F^{lone}	$X^{bridge}(Ag)$	$X^{bridge}(Cd)$	$X^{bridge}(Ag)$ / $X^{bridge}(Cd)$
F^-	Co	d^7	BS(Ag)	0.516	2.791	0.190	0.096	0.048	200%
	Ni	d^8	BS(Ag)	0.551	1.792	0.183	0.094	0.020	470%
	Cu	d^9	BS(Ag)	0.545	0.742	0.172	0.119	0.037	322%
	Nd	f^3	BS(Ln)	0.487	3.024	0.391	0.016	-0.004	-400%
	Pm	f^4	BS(Ag)	0.482	4.021	0.402	0.013	-0.004	-325%
	Sm	f^5	HS	0.482	5.033	0.404	0.009	-0.007	-129%
O^{2-}	Co	d^7	BS(Ag)*	0.382	2.818	0.024	0.484	0.140	346%
	Ni	d^8	HS	0.391	1.817	0.024	0.491	0.114	431%
	Cu	d^9	BS(Ag)*	0.437	0.757	0.063	0.124	0.037	335%
	Sm	f^5	BS(Ag)	0.522	5.039	0.170	0.106	-0.030	-353%
	Eu	f^6	BS(Ag)	0.519	6.051	0.172	0.104	-0.030	-347%
	Gd	f^7	BS(Ag)	0.524	7.023	0.171	0.119	-0.017	-700%
	Tb	f^8	BS(Ln)	0.519	6.006	0.167	0.140	-0.005	-2800%
	Dy	f^9	BS(Ln)	0.523	4.993	0.167	0.142	0.003	4733%
	Ho	f^{10}	HS	0.522	3.999	0.167	0.143	0.002	7150%
	Er	f^{11}	BS(Ag)	0.524	3.004	0.168	0.137	-0.001	-13700%
	Tm	f^{12}	HS	0.524	3.004	0.168	0.137	0.006	2283%
	Yb	f^{13}	HS	0.523	0.992	0.169	0.142	0.004	3550%
Cl^-	Co	d^7	BS(M)	0.498	2.773	0.190	0.138	0.042	329%
	Ni	d^8	BS(Ag)	0.551	1.792	0.183	0.094	0.020	470%
	Pr	f^2	BS(Ag)	0.482	2.033	0.346	0.044	-0.005	-880%
	Nd	f^3	BS(Ln)	0.483	3.028	0.345	0.044	-0.005	-880%
	Pm	f^4	BS(Ag)	0.480	4.023	0.351	0.042	-0.006	-700%
	Sm	f^5	BS(Ln)	0.479	5.041	0.354	0.040	-0.007	-571%
	Eu	f^6	HS	0.477	6.056	0.359	0.040	-0.006	-667%
	Gd	f^7	HS	0.477	7.016	0.365	0.037	-0.007	-529%
	Tb	f^8	HS	0.476	5.995	0.365	0.039	-0.005	-780%
	Dy	f^9	HS	0.476	4.984	0.367	0.041	-0.003	-1367%
	Ho	f^{10}	HS	0.477	3.990	0.368	0.041	-0.002	-2050%

Table S5. Computed data for several Cu analogues **(a)** of Ag systems **(b)**: valence electron configuration (v.e.), ground state configuration (GS), values of silver-metal and metal-metal superexchange constants before and after Cu(II)→Zn(II) substitution (J_{CuM}, J_{MM}(Cu) and J_{MM}(Zn), respectively) or correspondingly Ag(II)→Cd(II) substitution. Each system has one GS out of three possible spin-states: high-spin "HS", broken-symmetry with S(Cu) of opposite polarity "BS(Cu)", or broken-symmetry with S(M) of opposite polarity "BS(M)", correspondingly for Ag. Ratio is defined as Ratio=J_{MM}(Cu)/J_{MM}(Zn), while difference as Δ=J_{MM}(Cu)–J_{MM}(Zn), or correspondingly with Ag and Cd. Clusters in principle may lack symmetry elements thus J_{CuM} may be an average over two interactions (Cu–M^1 and Cu–M^2). Each copper cluster was optimized in a ground state electronic configuration, with 3d and 4f electrons' configurations in the excited states checked to be equivalent to GS **(c)**.

a) Copper clusters $[M_2CuF_8]^{2-}$ (M = Ni, Co), $[M_2CuF_7O]^{3-}$ (M = Ni, Co) and $[M_2CuF_7O]^{-}$ (M = Eu, Gd, Tb)

Ligand	Metal	v.e.	GS	J_{CuM} / cm^{-1}	J_{MM}(Cu) / cm^{-1}	J_{MM}(Zn) / cm^{-1}	Ratio	Δ / cm^{-1}
F^-	Co	d^7	BS(M)	–12.4	–15.4	–15.4	100%	0.0
	Ni	d^8	BS(M)	–34.0	–16.3	–15.2	108%	–1.2
O^{2-}	Co	d^7	BS(M)	69.1	–38.2	–34.7	110%	–3.5
	Ni	d^8	BS(M)	109.5	–122.8	–124.0	99%	1.2
	Eu	f^6	BS(Cu)	–9.34	0.124	0.167	74%	–0.043
	Gd	f^7	BS(Cu)	–3.64	–0.090	–0.073	124%	–0.017
	Tb	f^8	BS(Cu)	–1.25	–0.140	–0.099	141%	–0.041
	Dy	f^9	BS(M)	–0.76	–0.262	–0.205	128%	–0.058

b) Silver clusters $[M_2AgF_8]^{2-}$ (M = Ni, Co), $[M_2AgF_7O]^{3-}$ (M = Ni, Co) and $[M_2AgF_7O]^{-}$ (M = Eu, Gd, Tb)

Ligand	Metal	v.e.	GS	J_{AgM} / cm^{-1}	J_{MM}(Ag) / cm^{-1}	J_{MM}(Cd) / cm^{-1}	Ratio	Δ / cm^{-1}
F^-	Co	d^7	BS(M)	41.0	–17.3	–18.2	95%	0.9
	Ni	d^8	BS(Ag)	–44.4	–12.5	–11.1	112%	–1.4
O^{2-}	Co	d^7	HS*	326.5	–71.1	–31.1	229%	–40.0
	Ni	d^8	HS	409.1	1670.6	–100.5	–1662%	1771.1
	Eu	f^6	BS(Ag)	–22.60	–0.016	0.084	–19%	–0.100
	Gd	f^7	BS(Ag)	–12.54	–0.146	–0.119	123%	–0.027
	Tb	f^8	BS(M)	–4.30	–0.160	–0.130	123%	–0.030
	Dy	f^9	BS(M)	–1.47	–0.293	–0.197	148%	–0.096

c) Copper clusters total energy and Löwdin spin populations of each orbital (specified with m_l number) in 3d or 4f subshell

$[Co_2CuF_8]^{2-}$ spin state	Energy / eV	**Co1** d(z^2)	d(xz)	d(yz)	d(x^2–y^2)	d(xy)	**Co2** d(z^2)	d(xz)	d(yz)	d(x^2–y^2)	d(xy)
Zn, BS	-147053.225602	-0.91	-0.08	-0.40	-0.77	-0.56	0.92	0.08	0.36	0.76	0.59
Zn, HS	-147053.217071	0.91	0.08	0.40	0.77	0.56	0.92	0.08	0.37	0.76	0.59
Cu, HS	-143179.215293	0.90	0.09	0.40	0.78	0.56	0.92	0.08	0.37	0.76	0.59
Cu, BS(Cu)	-143179.219900	0.91	0.08	0.40	0.77	0.56	0.92	0.08	0.37	0.76	0.59
Cu, BS(M1)	-143179.226474	-0.91	-0.08	-0.40	-0.77	-0.56	0.92	0.08	0.37	0.76	0.59
Cu, BS(M2)	-143179.225781	0.90	0.08	0.40	0.78	0.56	-0.92	-0.08	-0.37	-0.76	-0.59

$[Ni_2CuF_8]^{2-}$ spin state	Energy / eV	**Ni1** d(z^2)	d(xz)	d(yz)	d(x^2–y^2)	d(xy)	**Ni2** d(z^2)	d(xz)	d(yz)	d(x^2–y^2)	d(xy)
Zn, BS	-154035.397390	-0.47	-0.02	-0.39	-0.52	-0.36	0.46	0.05	0.36	0.73	0.17
Zn, HS	-154035.393643	0.46	0.02	0.39	0.52	0.36	0.45	0.05	0.36	0.73	0.17
Cu, HS	-150161.393814	0.46	0.02	0.39	0.52	0.37	0.45	0.05	0.37	0.73	0.17
Cu, BS(Cu)	-150161.402199	0.46	0.02	0.39	0.52	0.36	0.45	0.05	0.36	0.73	0.17
Cu, BS(M1)	-150161.401659	-0.46	-0.02	-0.39	-0.53	-0.36	0.45	0.05	0.36	0.73	0.17
Cu, BS(M2)	-150161.402425	0.46	0.02	0.39	0.52	0.37	-0.45	-0.05	-0.36	-0.73	-0.17

$[Co_2CuF_7O]^{3-}$	Energy / eV			**Co1**					**Co2**		
spin state		$d(z^2)$	d(xz)	d(yz)	$d(x^2–y^2)$	d(xy)	$d(z^2)$	d(xz)	d(yz)	$d(x^2–y^2)$	d(xy)
Zn, BS	-146375.939795	-0.88	-0.16	-0.50	-0.70	-0.47	0.89	0.18	0.49	0.67	0.49
Zn, HS	-146375.920499	0.88	0.16	0.51	0.70	0.47	0.89	0.17	0.49	0.68	0.49
Cu, HS	-142502.048229	0.89	0.16	0.51	0.70	0.47	0.89	0.17	0.49	0.68	0.49
Cu, BS(Cu)	-142502.022638	0.88	0.16	0.51	0.70	0.47	0.89	0.17	0.49	0.68	0.50
Cu, BS(M1)	-142502.057222	-0.88	-0.16	-0.50	-0.70	-0.47	0.89	0.18	0.49	0.67	0.49
Cu, BS(M2)	-142502.056138	0.88	0.17	0.50	0.70	0.47	-0.89	-0.17	-0.49	-0.67	-0.49

$[Ni_2CuF_7O]^{3-}$	Energy / eV			**Ni1**					**Ni2**		
spin state		$d(z^2)$	d(xz)	d(yz)	$d(x^2–y^2)$	d(xy)	$d(z^2)$	d(xz)	d(yz)	$d(x^2–y^2)$	d(xy)
Zn, BS	-153358.081123	-0.33	-0.11	-0.73	-0.39	-0.20	0.31	0.13	0.71	0.39	0.21
Zn, HS	-153358.050511	0.33	0.11	0.73	0.39	0.20	0.31	0.13	0.72	0.39	0.21
Cu, HS	-149484.182358	0.33	0.11	0.73	0.39	0.20	0.31	0.14	0.72	0.39	0.21
Cu, BS(Cu)	-149484.155323	0.33	0.11	0.73	0.39	0.20	0.31	0.13	0.72	0.40	0.21
Cu, BS(M1)	-149484.199021	-0.33	-0.11	-0.73	-0.39	-0.20	0.31	0.14	0.71	0.39	0.21
Cu, BS(M2)	-149484.199311	0.33	0.11	0.72	0.39	0.20	-0.31	-0.13	-0.71	-0.39	-0.21

$[Eu_2CuF_7O]^{-}$	Energy / eV				**Eu1**							**Eu2**			
spin state		f0	f+1	f-1	f+2	f-2	f+3	f-3	f0	f+1	f-1	f+2	f-2	f+3	f-3
Zn, BS	-673148.050212	-0.97	-0.87	-0.47	-0.97	-0.96	-0.76	-0.97	0.96	0.64	0.70	0.96	0.99	0.87	0.85
Zn, HS	-673148.050583	0.97	0.87	0.47	0.97	0.97	0.75	0.97	0.96	0.64	0.70	0.96	0.99	0.88	0.84
Cu, HS	-669273.986836	0.97	0.87	0.47	0.97	0.96	0.76	0.97	0.96	0.64	0.70	0.96	0.99	0.88	0.84
Cu, BS(Cu)	-669273.993752	0.97	0.87	0.47	0.97	0.96	0.76	0.96	0.96	0.64	0.70	0.96	0.99	0.87	0.85
Cu, BS(M1)	-669273.990023	-0.97	-0.87	-0.47	-0.97	-0.96	-0.76	-0.96	0.96	0.64	0.70	0.96	0.99	0.87	0.85
Cu, BS(M2)	-669273.990016	0.97	0.87	0.47	0.97	0.96	0.76	0.96	-0.96	-0.64	-0.70	-0.96	-0.99	-0.87	-0.85

$[Gd_2CuF_7O]^{-}$	Energy / eV				**Gd1**							**Gd2**			
spin state		f0	f+1	f-1	f+2	f-2	f+3	f-3	f0	f+1	f-1	f+2	f-2	f+3	f-3
Zn, BS	-697339.551093	-0.99	-0.99	-0.99	-0.99	-0.99	-0.99	-0.99	0.99	0.99	0.99	0.99	0.99	0.99	0.99
Zn, HS	-697339.550872	0.99	0.99	0.99	0.99	0.99	0.99	0.99	0.99	0.99	0.99	0.99	0.99	0.99	0.99
Cu, HS	-693465.478415	0.99	0.99	0.99	0.99	0.99	0.99	0.99	0.99	0.99	0.99	0.99	0.99	0.99	0.99
Cu, BS(Cu)	-693465.481561	0.99	0.99	0.99	0.99	0.99	0.99	0.99	0.99	0.99	0.99	0.99	0.99	0.99	0.99
Cu, BS(M1)	-693465.480262	-0.99	-0.99	-0.99	-0.99	-0.99	-0.99	-0.99	0.99	0.99	0.99	0.99	0.99	0.99	0.99
Cu, BS(M2)	-693465.480260	0.99	0.99	0.99	0.99	0.99	0.99	0.99	-0.99	-0.99	-0.99	-0.99	-0.99	-0.99	-0.99

$[Tb_2CuF_7O]^{-}$	Energy / eV				**Tb1**							**Tb2**			
spin state		f0	f+1	f-1	f+2	f-2	f+3	f-3	f0	f+1	f-1	f+2	f-2	f+3	f-3
Zn, BS	-722134.249344	-0.97	-0.86	-0.56	-0.94	-0.96	-0.65	-0.98	0.99	0.97	0.40	0.99	0.98	0.96	0.65
Zn, HS	-722134.249124	0.97	0.86	0.56	0.95	0.96	0.65	0.98	0.99	0.97	0.40	0.99	0.98	0.96	0.65
Cu, HS	-718260.171907	0.97	0.88	0.57	0.93	0.94	0.66	0.98	0.99	0.97	0.42	0.99	0.95	0.96	0.65
Cu, BS(Cu)	-718260.172835	0.96	0.88	0.58	0.92	0.94	0.66	0.98	0.99	0.97	0.42	0.99	0.95	0.95	0.66
Cu, BS(M1)	-718260.172676	0.97	0.88	0.57	0.93	0.94	0.66	0.98	-0.99	-0.97	-0.42	-0.99	-0.95	-0.95	-0.66
Cu, BS(M2)	-718260.172688	-0.97	-0.88	-0.58	-0.93	-0.94	-0.66	-0.98	0.99	0.97	0.42	0.99	0.95	0.96	0.65

$[Dy_2CuF_7O]^{-}$	Energy / eV				**Dy1**							**Dy2**			
spin state		f0	f+1	f-1	f+2	f-2	f+3	f-3	f0	f+1	f-1	f+2	f-2	f+3	f-3
Zn, BS	-747581.408492	-0.94	-0.28	-0.54	-0.96	-0.97	-0.28	-0.95	0.94	0.52	0.32	0.98	0.94	0.82	0.42
Zn, HS	-747581.408176	0.95	0.28	0.54	0.96	0.97	0.28	0.95	0.94	0.52	0.32	0.98	0.94	0.82	0.42
Cu, HS	-743707.333236	0.93	0.29	0.54	0.96	0.97	0.28	0.95	0.94	0.52	0.32	0.98	0.94	0.82	0.42
Cu, BS(Cu)	-743707.333705	0.94	0.28	0.54	0.96	0.97	0.28	0.95	0.93	0.53	0.32	0.98	0.93	0.81	0.42
Cu, BS(M1)	-743707.333877	-0.93	-0.29	-0.54	-0.96	-0.97	-0.28	-0.95	0.94	0.52	0.32	0.98	0.94	0.82	0.42
Cu, BS(M2)	-743707.333874	0.93	0.29	0.54	0.96	0.97	0.28	0.95	-0.94	-0.52	-0.31	-0.98	-0.94	-0.82	-0.42

Table S6. Computed data for TEMPO analogues of molecules presented in **Figure 1** in the main article, of a formula [M_2F_4X·TEMPO]. All systems were ionically relaxed in their GS configuration. XYZ are provided in the 6th section of SI. The reference system used is a substitution of neutral TEMPO with an O^{2-} ligand. Ratio is defined as Ratio=J_{MM}(TEMPO)/J_{MM}(Ref.), while difference as Δ=J_{MM}(TEMPO)–J_{MM}(Ref.). Note that a considerable difference between X = F^- and O^{2-} for a Gd^{3+} cation arises from the geometry change between the two, i.e. with X = F^- we notice a rotation of TEMPO ring with respect to Gd_2XO plane, which causes rotation of 2p hole on O(TEMPO) out of Gd-O(TEMPO)-Gd plane. The J_{MM} with a TEMPO radical for a Ni system could not be obtained due to orbital configuration changes between a different spin states (i.e. for BS(M1) and BS(M2) states).

Ligand	Metal	v.e.	GS	$J_{TEMPO\text{-}M}$ / cm^{-1}	J_{MM}(TEMPO) / cm^{-1}	J_{MM}(Ref.) / cm^{-1}	Ratio	Δ / cm^{-1}
F^-	Ni	d^8	HS	228.8	-	224.9	-	-
	Gd	f^7	BS(M)	1.0	–0.14	–0.10	145%	–0.04
O^{2-}	Gd	f^7	HS	11.5	–0.39	0.54	–73%	–0.93

3. Oxide $[AgM_2F_7O]$ clusters

Table S7. Total energy and Löwdin spin populations of each orbital (specified with m_l number) in 3*d* or 4*f* subshell for each 3d TM or Ln cation in the clusters of $[AgM_2F_7O]$ type.

State	Energy / eV	Co1					Co2				
		d(z²)	d(xz)	d(yz)	d(x²–y²)	d(xy)	d(z²)	d(xz)	d(yz)	d(x²–y²)	d(xy)
Cd, BS	-251255.386310	-0.85	-0.19	-0.51	-0.76	-0.42	0.85	0.21	0.49	0.73	0.44
Cd, HS	-251255.369040	0.86	0.18	0.51	0.76	0.42	0.85	0.20	0.49	0.74	0.44
Ag, HS	-243639.667685	0.85	0.24	0.50	0.76	0.43	0.84	0.26	0.48	0.74	0.45
Ag, BS(Ag)	-243639.546773	0.86	0.16	0.50	0.76	0.43	0.85	0.19	0.48	0.74	0.46
Ag, BS(M1)	-243639.646739	-0.85	-0.17	-0.49	-0.75	-0.44	0.83	0.27	0.49	0.75	0.44
Ag, BS(M2)	-243639.646743	0.84	0.24	0.51	0.77	0.41	-0.84	-0.20	-0.47	-0.73	-0.46

State	Energy / eV	Ni1					Ni2				
		d(z²)	d(xz)	d(yz)	d(x²–y²)	d(xy)	d(z²)	d(xz)	d(yz)	d(x²–y²)	d(xy)
Cd, BS	-258237.494750	-0.27	-0.18	-0.69	-0.39	-0.24	0.24	0.22	0.67	0.39	0.24
Cd, HS	-258237.469930	0.26	0.18	0.70	0.39	0.24	0.24	0.22	0.68	0.39	0.24
Ag, HS	-250621.714866	0.26	0.20	0.68	0.40	0.25	0.24	0.24	0.66	0.40	0.26
Ag, BS(Ag)	-250621.613866	0.24	0.18	0.69	0.40	0.25	0.22	0.22	0.67	0.40	0.25
Ag, BS(M1)	-250621.251860	-0.25	-0.17	-0.68	-0.40	-0.25	0.24	0.24	0.65	0.40	0.25
Ag, BS(M2)	-250621.251886	0.26	0.20	0.67	0.40	0.25	-0.22	-0.21	-0.66	-0.40	-0.25

State	Energy / eV	Cu1					Cu2				
		d(z²)	d(xz)	d(yz)	d(x²–y²)	d(xy)	d(z²)	d(xz)	d(yz)	d(x²–y²)	d(xy)
Cd, BS	-265598.697108	-0.50	-0.06	-0.02	-0.03	-0.13	0.48	0.08	0.01	0.04	0.13
Cd, HS	-265598.673592	0.51	0.05	0.02	0.04	0.13	0.49	0.07	0.02	0.04	0.13
Ag, HS	-257982.945014	0.49	0.07	0.02	0.04	0.14	0.47	0.09	0.02	0.04	0.14
Ag, BS(Ag)	-257982.976526	0.50	0.05	0.02	0.04	0.14	0.48	0.07	0.02	0.04	0.13
Ag, BS(M1)	-257982.995891	-0.49	-0.06	-0.02	-0.03	-0.13	0.47	0.09	0.02	0.04	0.13
Ag, BS(M2)	-257983.000794	0.49	0.07	0.02	0.03	0.13	-0.47	-0.08	-0.02	-0.04	-0.13

State	Energy / eV	Sm1							Sm2						
		f0	f+1	f-1	f+2	f-2	f+3	f-3	f0	f+1	f-1	f+2	f-2	f+3	f-3
Cd, BS	-754456.534500	-0.53	-0.65	-0.84	-0.67	-0.83	-0.79	-0.67	0.66	0.45	0.88	0.65	0.92	0.54	0.89
Cd, HS	-754456.532929	0.53	0.65	0.84	0.67	0.82	0.78	0.68	0.66	0.45	0.87	0.65	0.92	0.53	0.89
Ag, HS	-746840.437068	0.53	0.65	0.84	0.67	0.82	0.78	0.68	0.66	0.45	0.87	0.65	0.92	0.53	0.89
Ag, BS(Ag)	-746840.455552	0.52	0.66	0.83	0.66	0.84	0.79	0.67	0.64	0.47	0.86	0.65	0.93	0.51	0.91
Ag, BS(M1)	-746840.448028	-0.52	-0.65	-0.84	-0.66	-0.84	-0.79	-0.67	0.66	0.45	0.88	0.65	0.92	0.54	0.89
Ag, BS(M2)	-746840.447903	0.54	0.65	0.84	0.67	0.82	0.79	0.68	-0.63	-0.49	-0.87	-0.64	-0.93	-0.51	-0.91

State	Energy / eV	Eu1							Eu2						
		f0	f+1	f-1	f+2	f-2	f+3	f-3	f0	f+1	f-1	f+2	f-2	f+3	f-3
Cd, BS	-778027.349127	-0.78	-0.89	-0.66	-0.98	-0.98	-0.72	-0.96	0.96	0.63	0.83	0.83	0.98	0.89	0.87
Cd, HS	-778027.349314	0.78	0.89	0.66	0.98	0.98	0.71	0.96	0.96	0.63	0.83	0.83	0.98	0.89	0.86
Ag, HS	-770411.260243	0.78	0.88	0.66	0.98	0.98	0.72	0.97	0.96	0.63	0.83	0.83	0.98	0.89	0.87
Ag, BS(Ag)	-770411.276986	0.78	0.89	0.66	0.98	0.98	0.72	0.95	0.96	0.63	0.83	0.83	0.98	0.88	0.87
Ag, BS(M1)	-770411.268655	-0.78	-0.89	-0.66	-0.98	-0.98	-0.72	-0.95	0.96	0.63	0.83	0.83	0.98	0.89	0.87
Ag, BS(M2)	-770411.268644	0.78	0.89	0.66	0.98	0.98	0.72	0.96	-0.95	-0.63	-0.83	-0.83	-0.98	-0.87	-0.88

State	Energy / eV	Gd1							Gd2						
		f0	f+1	f-1	f+2	f-2	f+3	f-3	f0	f+1	f-1	f+2	f-2	f+3	f-3
Cd, BS	-802215.882009	-0.99	-0.99	-0.99	-0.99	-0.99	-0.99	-0.99	0.99	0.99	0.99	0.99	0.99	0.99	0.99
Cd, HS	-802215.881650	0.99	0.99	0.99	0.99	0.99	0.99	0.99	0.99	0.99	0.99	0.99	0.99	0.99	0.99
Ag, HS	-794597.616837	0.99	0.99	0.99	0.99	0.99	0.99	0.99	0.99	0.99	0.99	0.99	0.99	0.99	0.99
Ag, BS(Ag)	-794597.627676	0.99	0.99	0.99	0.99	0.99	0.99	0.99	0.99	0.99	0.99	0.99	0.99	0.99	0.99
Ag, BS(M1)	-794597.622703	-0.99	-0.99	-0.99	-0.99	-0.99	-0.99	-0.99	0.99	0.99	0.99	0.99	0.99	0.99	0.99
Ag, BS(M2)	-794597.622693	0.99	0.99	0.99	0.99	0.99	0.99	0.99	-0.99	-0.99	-0.99	-0.99	-0.99	-0.99	-0.99

State	Energy / eV	Tb1							Tb2						
		f0	f+1	f-1	f+2	f-2	f+3	f-3	f0	f+1	f-1	f+2	f-2	f+3	f-3
Cd, BS	-827019.084945	-0.99	-0.81	-0.55	-0.99	-0.99	-0.61	-0.99	0.94	0.52	0.91	0.98	0.99	0.83	0.75
Cd, HS	-827019.084656	0.99	0.81	0.55	0.99	0.99	0.61	0.99	0.94	0.52	0.91	0.99	0.99	0.83	0.75
Ag, HS	-819402.974393	0.98	0.84	0.55	0.98	0.98	0.63	0.98	0.94	0.51	0.92	0.98	0.99	0.79	0.80
Ag, BS(Ag)	-819402.977579	0.98	0.82	0.57	0.97	0.98	0.62	0.99	0.94	0.52	0.91	0.99	0.99	0.82	0.75
Ag, BS(M1)	-819402.978848	0.99	0.84	0.54	0.98	0.98	0.62	0.98	-0.94	-0.52	-0.91	-0.99	-0.99	-0.82	-0.75
Ag, BS(M2)	-819402.973835	-0.98	-0.82	-0.56	-0.97	-0.98	-0.62	-0.99	0.94	0.51	0.92	0.98	0.99	0.79	0.80

State	Energy / eV	Dy1							Dy2						
		f0	f+1	f-1	f+2	f-2	f+3	f-3	f0	f+1	f-1	f+2	f-2	f+3	f-3
Cd, BS	-852460.708168	-0.94	-0.25	-0.56	-0.96	-0.98	-0.30	-0.94	0.93	0.49	0.34	0.98	0.94	0.75	0.49
Cd, HS	-852460.707864	0.95	0.25	0.56	0.96	0.98	0.30	0.94	0.93	0.49	0.34	0.98	0.94	0.75	0.50
Ag, HS	-844844.595358	0.94	0.26	0.56	0.96	0.97	0.30	0.94	0.93	0.49	0.34	0.98	0.94	0.77	0.48
Ag, BS(Ag)	-844844.596262	0.94	0.25	0.56	0.96	0.98	0.30	0.94	0.93	0.49	0.34	0.98	0.94	0.75	0.49
Ag, BS(M1)	-844844.596332	-0.94	-0.25	-0.56	-0.96	-0.98	-0.30	-0.94	0.93	0.49	0.34	0.98	0.94	0.77	0.48
Ag, BS(M2)	-844844.596193	0.94	0.26	0.56	0.96	0.97	0.30	0.94	-0.93	-0.49	-0.34	-0.98	-0.94	-0.75	-0.49

State	Energy / eV	Ho1							Ho2						
		f0	f+1	f-1	f+2	f-2	f+3	f-3	f0	f+1	f-1	f+2	f-2	f+3	f-3
Cd, BS	-878550.475829	0.69	0.24	0.72	0.66	0.53	0.19	0.93	-0.67	-0.13	-0.84	-0.25	-0.93	-0.87	-0.25
Cd, HS	-878550.475715	0.69	0.24	0.72	0.67	0.52	0.19	0.93	0.67	0.13	0.84	0.25	0.93	0.87	0.25
Ag, HS	-870934.360557	0.69	0.21	0.75	0.58	0.60	0.25	0.87	0.68	0.14	0.84	0.22	0.95	0.92	0.20
Ag, BS(Ag)	-870934.359151	0.69	0.23	0.73	0.65	0.54	0.19	0.92	0.68	0.13	0.84	0.24	0.94	0.88	0.23
Ag, BS(M1)	-870934.360126	-0.69	-0.25	-0.71	-0.63	-0.56	-0.19	-0.92	0.68	0.14	0.84	0.22	0.95	0.93	0.20
Ag, BS(M2)	-870934.360136	0.69	0.21	0.75	0.57	0.61	0.25	0.87	-0.68	-0.13	-0.84	-0.24	-0.94	-0.89	-0.23

State	Energy / eV	Er1							Er2						
		f0	f+1	f-1	f+2	f-2	f+3	f-3	f0	f+1	f-1	f+2	f-2	f+3	f-3
Cd, BS	-905298.013061	-0.24	-0.34	-0.78	-0.23	-0.52	-0.03	-0.83	0.27	0.55	0.52	0.07	0.69	0.58	0.27
Cd, HS	-905298.012988	0.24	0.34	0.78	0.24	0.51	0.03	0.83	0.27	0.55	0.52	0.07	0.69	0.58	0.28
Ag, HS	-897673.739213	0.23	0.35	0.77	0.21	0.53	0.03	0.83	0.27	0.56	0.51	0.08	0.69	0.61	0.24
Ag, BS(Ag)	-897673.742544	0.24	0.34	0.78	0.24	0.51	0.03	0.82	0.27	0.55	0.52	0.07	0.69	0.57	0.28
Ag, BS(M1)	-897673.741049	-0.24	-0.34	-0.78	-0.24	-0.51	-0.03	-0.82	0.27	0.56	0.51	0.08	0.69	0.62	0.24
Ag, BS(M2)	-897673.741028	0.23	0.35	0.77	0.21	0.54	0.03	0.83	-0.27	-0.55	-0.52	-0.07	-0.69	-0.57	-0.28

State	Energy / eV	Tm1							Tm2						
		f0	f+1	f-1	f+2	f-2	f+3	f-3	f0	f+1	f-1	f+2	f-2	f+3	f-3
Cd, BS	-932714.187618	-0.06	-0.41	-0.80	-0.05	-0.04	-0.21	-0.41	0.04	0.46	0.78	0.05	0.01	0.47	0.16
Cd, HS	-932714.187414	0.03	0.44	0.81	0.03	0.02	0.22	0.41	0.04	0.45	0.78	0.05	0.01	0.47	0.15
Ag, HS	-925098.065351	0.03	0.43	0.82	0.03	0.02	0.22	0.41	0.04	0.44	0.80	0.05	0.01	0.48	0.14
Ag, BS(Ag)	-925098.059992	0.03	0.44	0.82	0.03	0.02	0.22	0.40	0.04	0.45	0.78	0.05	0.01	0.47	0.15
Ag, BS(M1)	-925098.062883	-0.02	-0.46	-0.82	-0.02	-0.02	-0.22	-0.41	0.04	0.44	0.80	0.05	0.01	0.48	0.14
Ag, BS(M2)	-925098.062901	0.04	0.42	0.82	0.03	0.03	0.22	0.41	-0.03	-0.48	-0.78	-0.04	-0.01	-0.47	-0.15

State	Energy / eV	Yb1							Yb2						
		f0	f+1	f-1	f+2	f-2	f+3	f-3	f0	f+1	f-1	f+2	f-2	f+3	f-3
Cd, BS	-960812.676145	-0.02	-0.13	-0.49	-0.02	-0.02	-0.30	0.00	0.03	0.33	0.27	0.03	0.00	0.09	0.22
Cd, HS	-960812.676179	0.02	0.13	0.49	0.02	0.02	0.30	0.00	0.03	0.33	0.27	0.03	0.00	0.09	0.22
Ag, HS	-953196.552443	0.02	0.12	0.50	0.02	0.02	0.29	0.01	0.03	0.32	0.29	0.03	0.00	0.11	0.19
Ag, BS(Ag)	-953196.550022	0.02	0.13	0.49	0.02	0.02	0.29	0.00	0.03	0.34	0.28	0.03	0.00	0.09	0.21
Ag, BS(M1)	-953196.551208	-0.02	-0.12	-0.49	-0.02	-0.02	-0.29	0.00	0.03	0.32	0.29	0.03	0.00	0.11	0.19
Ag, BS(M2)	-953196.551217	0.02	0.12	0.50	0.02	0.02	0.29	0.02	-0.03	-0.34	-0.28	-0.03	0.00	-0.09	-0.21

Table S8. Total energy and Löwdin spin populations of each orbital (specified with m_l number) in 4*s*, 4*p* and 3*d* subshells for the silver cation in the clusters of $[AgM_2F_7O]^-$ type.

State	Energy / eV	Co								
		s	p_z	p_x	p_y	$d(z^2)$	d(xz)	d(yz)	$d(x^2–y^2)$	d(xy)
Ag, HS	-243639.667685	0.030	0.000	-0.002	-0.004	0.227	0.001	0.007	0.123	0.000
Ag, BS(Ag)	-243639.546773	-0.027	0.009	0.002	0.000	-0.258	0.001	-0.008	-0.144	0.000
Ag, BS(M1)	-243639.646739	0.029	-0.004	-0.002	-0.002	0.234	0.000	0.008	0.130	0.000
Ag, BS(M2)	-243639.646743	0.029	-0.004	-0.002	-0.002	0.234	0.001	0.008	0.129	0.000

State	Energy / eV	Ni								
		s	p_z	p_x	p_y	$d(z^2)$	d(xz)	d(yz)	$d(x^2–y^2)$	d(xy)
Ag, HS	-250621.714866	0.031	-0.001	-0.003	-0.004	0.239	0.000	0.000	0.095	0.034
Ag, BS(Ag)	-250621.613866	-0.030	0.008	0.002	0.001	-0.263	-0.001	0.000	-0.103	-0.037
Ag, BS(M1)	-250621.251860	0.030	-0.005	-0.003	-0.002	0.250	0.001	0.000	0.098	0.036
Ag, BS(M2)	-250621.251886	0.030	-0.005	-0.003	-0.002	0.250	0.000	0.000	0.099	0.036

State	Energy / eV	Cu								
		s	p_z	p_x	p_y	$d(z^2)$	d(xz)	d(yz)	$d(x^2–y^2)$	d(xy)
Ag, HS	-257982.945014	0.012	-0.002	-0.003	-0.004	0.297	0.000	0.001	0.136	0.000
Ag, BS(Ag)	-257982.976526	-0.015	0.011	0.002	0.001	-0.293	0.000	-0.001	-0.135	0.000
Ag, BS(M1)	-257982.995891	0.013	-0.006	-0.003	-0.003	0.293	0.000	0.001	0.135	0.000
Ag, BS(M2)	-257983.000794	0.013	-0.006	-0.003	-0.003	0.292	0.001	0.001	0.134	0.000

State	Energy / eV	Sm								
		s	p_z	p_x	p_y	$d(z^2)$	d(xz)	d(yz)	$d(x^2–y^2)$	d(xy)
Ag, HS	-746840.437068	0.029	-0.001	-0.005	-0.005	0.003	0.020	0.069	0.337	0.075
Ag, BS(Ag)	-746840.455552	-0.029	0.002	0.004	0.005	-0.003	-0.019	-0.068	-0.335	-0.074
Ag, BS(M1)	-746840.448028	0.029	-0.002	-0.005	-0.005	0.003	0.019	0.069	0.336	0.075
Ag, BS(M2)	-746840.447903	0.029	-0.002	-0.005	-0.005	0.003	0.019	0.069	0.336	0.075

State	Energy / eV	Eu								
		s	p_z	p_x	p_y	$d(z^2)$	d(xz)	d(yz)	$d(x^2–y^2)$	d(xy)
Ag, HS	-770411.260243	0.028	-0.001	-0.005	-0.005	0.008	0.010	0.028	0.386	0.070
Ag, BS(Ag)	-770411.276986	-0.029	0.001	0.004	0.005	-0.008	-0.010	-0.027	-0.384	-0.071
Ag, BS(M1)	-770411.268655	0.028	-0.001	-0.005	-0.005	0.008	0.010	0.028	0.385	0.070
Ag, BS(M2)	-770411.268644	0.028	-0.001	-0.005	-0.005	0.008	0.010	0.028	0.384	0.071

State	Energy / eV	Gd								
		s	p_z	p_x	p_y	$d(z^2)$	d(xz)	d(yz)	$d(x^2–y^2)$	d(xy)
Ag, HS	-794597.616837	0.031	-0.001	-0.005	-0.005	0.015	0.004	0.001	0.418	0.066
Ag, BS(Ag)	-794597.627676	-0.031	0.001	0.004	0.006	-0.015	-0.004	0.000	-0.416	-0.064
Ag, BS(M1)	-794597.622703	0.031	-0.001	-0.004	-0.006	0.015	0.004	0.000	0.417	0.064
Ag, BS(M2)	-794597.622693	0.031	-0.001	-0.005	-0.005	0.015	0.004	0.000	0.417	0.065

State	Energy / eV	Tb								
		s	p_z	p_x	p_y	$d(z^2)$	d(xz)	d(yz)	$d(x^2–y^2)$	d(xy)
Ag, HS	-819402.974393	0.032	-0.001	-0.005	-0.005	0.015	0.004	0.001	0.407	0.073
Ag, BS(Ag)	-819402.977579	-0.032	0.001	0.004	0.006	-0.015	-0.004	0.000	-0.410	-0.071
Ag, BS(M1)	-819402.978848	0.032	-0.001	-0.005	-0.005	0.015	0.004	0.000	0.407	0.072
Ag, BS(M2)	-819402.973835	0.032	-0.001	-0.004	-0.005	0.015	0.004	0.000	0.410	0.071

State	Energy / eV	Dy								
		s	p_z	p_x	p_y	$d(z^2)$	d(xz)	d(yz)	$d(x^2–y^2)$	d(xy)
Ag, HS	-844844.595358	0.032	-0.001	-0.005	-0.005	0.015	0.004	0.001	0.417	0.065
Ag, BS(Ag)	-844844.596262	-0.033	0.001	0.004	0.006	-0.015	-0.004	0.000	-0.417	-0.064
Ag, BS(M1)	-844844.596332	0.033	-0.001	-0.004	-0.006	0.015	0.004	0.000	0.418	0.064
Ag, BS(M2)	-844844.596193	0.033	-0.001	-0.005	-0.005	0.015	0.004	0.000	0.416	0.065

State	Energy / eV	Ho								
		s	p_z	p_x	p_y	$d(z^2)$	d(xz)	d(yz)	$d(x^2–y^2)$	d(xy)
Ag, HS	-870934.360557	0.033	-0.001	-0.004	-0.005	0.015	0.004	0.000	0.418	0.063
Ag, BS(Ag)	-870934.359151	0.033	-0.001	-0.004	-0.005	0.015	0.004	0.000	0.416	0.065
Ag, BS(M1)	-870934.360126	-0.033	0.001	0.004	0.005	-0.015	-0.004	0.000	-0.418	-0.064
Ag, BS(M2)	-870934.360136	0.033	-0.001	-0.004	-0.005	0.015	0.004	0.000	0.419	0.064

State	Energy / eV	Er								
		s	p_z	p_x	p_y	$d(z^2)$	d(xz)	d(yz)	$d(x^2–y^2)$	d(xy)
Ag, HS	-897673.739213	0.034	-0.001	-0.004	-0.005	0.016	0.004	0.000	0.417	0.064
Ag, BS(Ag)	-897673.742544	-0.034	0.001	0.004	0.006	-0.016	-0.004	0.000	-0.417	-0.064
Ag, BS(M1)	-897673.741049	0.034	-0.001	-0.004	-0.005	0.016	0.004	0.000	0.417	0.063
Ag, BS(M2)	-897673.741028	0.034	-0.001	-0.004	-0.005	0.016	0.004	0.000	0.416	0.064

State	Energy / eV	Tm								
		s	p_z	p_x	p_y	$d(z^2)$	d(xz)	d(yz)	$d(x^2–y^2)$	d(xy)
Ag, HS	-925098.065351	0.035	-0.001	-0.004	-0.005	0.016	0.003	0.000	0.415	0.064
Ag, BS(Ag)	-925098.059992	-0.036	0.001	0.004	0.006	-0.016	-0.003	0.000	-0.416	-0.063
Ag, BS(M1)	-925098.062883	0.035	-0.001	-0.004	-0.005	0.016	0.003	0.000	0.415	0.063
Ag, BS(M2)	-925098.062901	0.035	-0.001	-0.004	-0.005	0.016	0.003	0.000	0.415	0.064

State	Energy / eV	Yb								
		s	p_z	p_x	p_y	$d(z^2)$	d(xz)	d(yz)	$d(x^2–y^2)$	d(xy)
Ag, HS	-953196.552443	0.035	-0.001	-0.004	-0.005	0.016	0.004	0.000	0.416	0.063
Ag, BS(Ag)	-953196.550022	-0.035	0.001	0.005	0.006	-0.016	-0.004	0.000	-0.416	-0.061
Ag, BS(M1)	-953196.551208	0.035	-0.001	-0.004	-0.005	0.016	0.004	0.000	0.416	0.062
Ag, BS(M2)	-953196.551217	0.035	-0.001	-0.004	-0.005	0.016	0.004	0.000	0.416	0.062

4. Chloride [AgM_2F_7Cl] clusters

Table S9. Total energy and Löwdin spin populations of each orbital (specified with m_l number) in 3*d* or 4*f* subshell for each 3d TM or Ln cation in the clusters of [AgM_2F_7Cl] type.

State	Energy / eV	Co1					Co2				
		d(z²)	d(xz)	d(yz)	d(x²–y²)	d(xy)	d(z²)	d(xz)	d(yz)	d(x²–y²)	d(xy)
Cd, BS	-261795.059002	-0.65	-0.27	-0.53	-0.88	-0.37	0.69	0.23	0.50	0.88	0.40
Cd, HS	-261795.041490	0.65	0.28	0.53	0.88	0.37	0.69	0.23	0.50	0.88	0.39
Ag, HS	-254179.004796	0.66	0.28	0.53	0.88	0.37	0.70	0.23	0.50	0.88	0.39
Ag, BS(Ag)	-254179.001954	0.66	0.27	0.53	0.88	0.36	0.70	0.22	0.51	0.88	0.39
Ag, BS(M1)	-254179.022114	-0.66	-0.26	-0.53	-0.88	-0.36	0.70	0.23	0.50	0.88	0.39
Ag, BS(M2)	-254179.020566	0.66	0.28	0.53	0.88	0.37	-0.70	-0.22	-0.51	-0.88	-0.39

State	Energy / eV	Ni1					Ni2				
		d(z²)	d(xz)	d(yz)	d(x²–y²)	d(xy)	d(z²)	d(xz)	d(yz)	d(x²–y²)	d(xy)
Cd, BS	-268777.332263	-0.07	-0.12	-0.79	-0.56	-0.21	0.07	0.10	0.79	0.58	0.21
Cd, HS	-268777.331983	0.07	0.12	0.79	0.56	0.21	0.07	0.10	0.79	0.58	0.21
Ag, HS	-261161.271996	0.06	0.13	0.80	0.56	0.21	0.07	0.11	0.79	0.57	0.21
Ag, BS(Ag)	-261161.292511	0.06	0.12	0.79	0.56	0.21	0.07	0.10	0.79	0.58	0.21
Ag, BS(M1)	-261161.282920	-0.06	-0.12	-0.79	-0.56	-0.21	0.07	0.11	0.79	0.57	0.21
Ag, BS(M2)	-261161.282898	0.06	0.13	0.80	0.56	0.21	-0.07	-0.10	-0.79	-0.58	-0.21

State	Energy / eV	Pr1							Pr2						
		f0	f+1	f-1	f+2	f-2	f+3	f-3	f0	f+1	f-1	f+2	f-2	f+3	f-3
Cd, BS	-697872.6545164	-0.03	-0.51	-0.57	-0.02	-0.04	-0.17	-0.65	0.04	0.25	0.82	0.03	0.06	0.30	0.52
Cd, HS	-697872.6544338	0.03	0.51	0.57	0.02	0.04	0.18	0.64	0.04	0.25	0.82	0.03	0.06	0.30	0.52
Ag, HS	-690256.6194200	0.03	0.51	0.57	0.02	0.04	0.19	0.64	0.04	0.24	0.82	0.03	0.06	0.29	0.52
Ag, BS(Ag)	-690256.6271823	0.03	0.51	0.57	0.02	0.04	0.19	0.64	0.04	0.24	0.82	0.02	0.06	0.29	0.53
Ag, BS(M1)	-690256.6233938	-0.03	-0.51	-0.57	-0.02	-0.04	-0.19	-0.64	0.04	0.24	0.82	0.03	0.06	0.29	0.52
Ag, BS(M2)	-690256.6234035	0.03	0.51	0.57	0.02	0.04	0.18	0.64	-0.04	-0.24	-0.82	-0.02	-0.06	-0.29	-0.53

State	Energy / eV	Nd1							Nd2						
		f0	f+1	f-1	f+2	f-2	f+3	f-3	f0	f+1	f-1	f+2	f-2	f+3	f-3
Cd, BS	-719654.7933352	-0.22	-0.46	-0.61	-0.06	-0.72	-0.25	-0.67	0.28	0.70	0.27	0.59	0.27	0.41	0.46
Cd, HS	-719654.7932265	0.22	0.46	0.61	0.06	0.72	0.25	0.67	0.28	0.70	0.27	0.59	0.27	0.40	0.46
Ag, HS	-712038.7522503	0.22	0.46	0.61	0.05	0.72	0.27	0.65	0.28	0.71	0.27	0.58	0.27	0.41	0.46
Ag, BS(Ag)	-712038.7539078	0.22	0.46	0.60	0.05	0.72	0.29	0.63	0.27	0.72	0.27	0.58	0.27	0.40	0.47
Ag, BS(M1)	-712038.7546793	-0.22	-0.47	-0.60	-0.05	-0.72	-0.29	-0.63	0.28	0.71	0.27	0.58	0.27	0.41	0.46
Ag, BS(M2)	-712038.7516820	0.22	0.46	0.61	0.05	0.72	0.27	0.65	-0.27	-0.71	-0.27	-0.58	-0.27	-0.40	-0.47

State	Energy / eV	Pm1							Pm2						
		f0	f+1	f-1	f+2	f-2	f+3	f-3	f0	f+1	f-1	f+2	f-2	f+3	f-3
Cd, BS	-742023.1463532	-0.69	-0.48	-0.46	-0.95	-0.27	-0.51	-0.62	0.67	0.25	0.71	0.56	0.65	0.29	0.84
Cd, HS	-742023.1460812	0.69	0.48	0.46	0.95	0.27	0.51	0.62	0.67	0.25	0.71	0.56	0.65	0.29	0.84
Ag, HS	-734407.1112579	0.69	0.48	0.46	0.95	0.27	0.51	0.62	0.67	0.25	0.71	0.56	0.65	0.29	0.84
Ag, BS(Ag)	-734407.1122200	0.69	0.47	0.46	0.95	0.27	0.51	0.62	0.67	0.25	0.71	0.56	0.65	0.29	0.84
Ag, BS(M1)	-734407.1120134	-0.69	-0.48	-0.46	-0.95	-0.27	-0.52	-0.61	0.67	0.25	0.71	0.56	0.64	0.29	0.84
Ag, BS(M2)	-734407.1120158	0.69	0.48	0.45	0.95	0.27	0.52	0.61	-0.67	-0.25	-0.71	-0.56	-0.65	-0.29	-0.84

State	Energy / eV	Sm1							Sm2						
		f0	f+1	f-1	f+2	f-2	f+3	f-3	f0	f+1	f-1	f+2	f-2	f+3	f-3
Cd, BS	-764986.8688465	-0.89	-0.53	-0.68	-0.88	-0.47	-0.99	-0.53	0.94	0.40	0.33	0.97	0.95	0.83	0.55
Cd, HS	-764986.8686585	0.90	0.54	0.68	0.88	0.44	0.99	0.54	0.94	0.40	0.33	0.97	0.95	0.83	0.54
Ag, HS	-757370.8307209	0.90	0.54	0.68	0.88	0.44	0.99	0.54	0.94	0.40	0.33	0.97	0.95	0.83	0.55
Ag, BS(Ag)	-757370.8294215	0.90	0.53	0.69	0.87	0.47	0.99	0.53	0.94	0.40	0.33	0.97	0.95	0.83	0.55
Ag, BS(M1)	-757370.8303880	-0.90	-0.54	-0.69	-0.88	-0.43	-0.99	-0.56	0.94	0.40	0.33	0.97	0.95	0.83	0.55
Ag, BS(M2)	-757370.8300333	0.90	0.53	0.68	0.87	0.48	0.99	0.53	-0.94	-0.40	-0.33	-0.97	-0.95	-0.83	-0.55

State	Energy / eV	Eu1							Eu2						
		f0	f+1	f-1	f+2	f-2	f+3	f-3	f0	f+1	f-1	f+2	f-2	f+3	f-3
Cd, BS	-788557.6839468	-0.94	-0.61	-0.70	-0.98	-0.99	-0.98	-0.78	0.94	0.39	0.92	0.98	0.99	0.85	0.91
Cd, HS	-788557.6837695	0.94	0.61	0.70	0.98	0.99	0.98	0.78	0.94	0.39	0.92	0.98	0.99	0.85	0.91
Ag, HS	-780941.6451370	0.94	0.61	0.70	0.98	0.99	0.98	0.78	0.94	0.39	0.92	0.98	0.99	0.86	0.91
Ag, BS(Ag)	-780941.6429831	0.94	0.60	0.71	0.98	0.99	0.99	0.78	0.94	0.39	0.92	0.98	0.99	0.85	0.91
Ag, BS(M1)	-780941.6442593	-0.94	-0.60	-0.71	-0.98	-0.99	-0.99	-0.78	0.94	0.39	0.92	0.98	0.99	0.86	0.91
Ag, BS(M2)	-780941.6442483	0.94	0.61	0.70	0.98	0.99	0.98	0.78	-0.94	-0.39	-0.92	-0.98	-0.99	-0.85	-0.92

State	Energy / eV	Gd1							Gd2						
		f0	f+1	f-1	f+2	f-2	f+3	f-3	f0	f+1	f-1	f+2	f-2	f+3	f-3
Cd, BS	-812749.0477548	-0.99	-0.99	-0.99	-0.99	-0.99	-0.99	-0.99	0.99	0.98	0.99	0.99	0.99	0.99	0.99
Cd, HS	-812749.0474756	0.99	0.99	0.99	0.99	0.99	0.99	0.99	0.99	0.98	0.99	0.99	0.99	0.99	0.99
Ag, HS	-805132.9916182	0.99	0.99	0.99	0.99	0.99	0.99	0.99	0.99	0.98	0.99	0.99	0.99	0.99	0.99
Ag, BS(Ag)	-805132.9897960	0.99	0.99	0.99	0.99	0.99	0.99	0.99	0.99	0.98	0.99	0.99	0.99	0.99	0.99
Ag, BS(M1)	-805132.9909971	-0.99	-0.99	-0.99	-0.99	-0.99	-0.99	-0.99	0.99	0.98	0.99	0.99	0.99	0.99	0.99
Ag, BS(M2)	-805132.9909952	0.99	0.99	0.99	0.99	0.99	0.99	0.99	-0.99	-0.98	-0.99	-0.99	-0.99	-0.99	-0.99

State	Energy / eV	Tb1							Tb2						
		f0	f+1	f-1	f+2	f-2	f+3	f-3	f0	f+1	f-1	f+2	f-2	f+3	f-3
Cd, BS	-837549.2105632	-0.98	-0.93	-0.52	-0.98	-0.95	-0.81	-0.75	0.95	0.72	0.70	0.98	0.98	0.81	0.77
Cd, HS	-837549.2103629	0.98	0.93	0.52	0.98	0.95	0.81	0.75	0.95	0.72	0.70	0.98	0.98	0.81	0.77
Ag, HS	-829933.1530550	0.98	0.93	0.52	0.97	0.95	0.80	0.76	0.96	0.72	0.71	0.97	0.99	0.84	0.75
Ag, BS(Ag)	-829933.1494068	0.98	0.93	0.52	0.97	0.95	0.80	0.76	0.96	0.72	0.70	0.97	0.99	0.82	0.76
Ag, BS(M1)	-829933.1524994	-0.98	-0.93	-0.52	-0.98	-0.95	-0.80	-0.76	0.96	0.72	0.71	0.97	0.99	0.83	0.75
Ag, BS(M2)	-829933.1503849	0.98	0.93	0.53	0.97	0.95	0.80	0.76	-0.96	-0.72	-0.70	-0.97	-0.99	-0.82	-0.76

State	Energy / eV	Dy1							Dy2						
		f0	f+1	f-1	f+2	f-2	f+3	f-3	f0	f+1	f-1	f+2	f-2	f+3	f-3
Cd, BS	-862990.7620693	-0.94	-0.44	-0.44	-0.97	-0.94	-0.69	-0.51	0.95	0.67	0.19	0.97	0.94	0.82	0.37
Cd, HS	-862990.7619604	0.94	0.44	0.44	0.97	0.94	0.69	0.51	0.95	0.67	0.19	0.97	0.94	0.82	0.37
Ag, HS	-855374.6983829	0.94	0.44	0.44	0.97	0.94	0.68	0.52	0.95	0.68	0.18	0.97	0.94	0.83	0.37
Ag, BS(Ag)	-855374.6941362	0.94	0.44	0.44	0.97	0.94	0.68	0.52	0.95	0.68	0.18	0.97	0.94	0.83	0.37
Ag, BS(M1)	-855374.6963800	-0.94	-0.44	-0.44	-0.97	-0.94	-0.68	-0.52	0.95	0.68	0.18	0.97	0.94	0.83	0.37
Ag, BS(M2)	-855374.6963801	0.94	0.44	0.44	0.97	0.94	0.68	0.52	-0.96	-0.67	-0.18	-0.97	-0.94	-0.83	-0.37

State	Energy / eV	Ho1							Ho2						
		f0	f+1	f-1	f+2	f-2	f+3	f-3	f0	f+1	f-1	f+2	f-2	f+3	f-3
Cd, BS	-889080.4625254	-0.67	-0.16	-0.85	-0.28	-0.86	-0.78	-0.34	0.59	0.43	0.70	0.29	0.81	0.63	0.49
Cd, HS	-889080.4624879	0.67	0.16	0.85	0.28	0.86	0.78	0.33	0.59	0.43	0.70	0.29	0.81	0.63	0.49
Ag, HS	-881464.3917648	0.68	0.15	0.85	0.28	0.86	0.78	0.34	0.59	0.43	0.70	0.28	0.82	0.64	0.48
Ag, BS(Ag)	-881464.3908812	0.67	0.16	0.85	0.28	0.87	0.78	0.33	0.59	0.44	0.70	0.29	0.81	0.63	0.49
Ag, BS(M1)	-881464.3913977	-0.68	-0.16	-0.85	-0.28	-0.87	-0.78	-0.33	0.59	0.43	0.70	0.28	0.82	0.64	0.48
Ag, BS(M2)	-881464.3913346	0.67	0.16	0.85	0.28	0.86	0.78	0.34	-0.59	-0.44	-0.69	-0.29	-0.81	-0.63	-0.49

Table S10. Total energy and Löwdin spin populations of each orbital (specified with m_l number) in 4*s*, 4*p* and 3*d* subshells for the silver cation in the clusters of [AgM_2F_7Cl] type.

State	Energy / eV	Co								
		s	p_z	p_x	p_y	$d(z^2)$	d(xz)	d(yz)	$d(x^2–y^2)$	d(xy)
Ag, HS	-254179.004796	-0.016	-0.005	-0.003	-0.003	0.364	0.000	0.013	0.148	0.000
Ag, BS(Ag)	-254179.001954	0.018	0.007	0.004	0.001	-0.367	0.000	-0.014	-0.150	0.000
Ag, BS(M1)	-254179.022114	-0.017	-0.006	-0.003	-0.002	0.365	0.000	0.014	0.149	0.000
Ag, BS(M2)	-254179.020566	-0.017	-0.006	-0.003	-0.002	0.366	0.000	0.014	0.149	0.000

State	Energy / eV	Ni								
		s	p_z	p_x	p_y	$d(z^2)$	d(xz)	d(yz)	$d(x^2–y^2)$	d(xy)
Ag, HS	-261161.271996	-0.005	-0.005	-0.005	-0.002	0.381	0.001	0.000	0.113	0.039
Ag, BS(Ag)	-261161.292511	0.010	0.007	0.005	0.001	-0.380	0.000	0.000	-0.112	-0.038
Ag, BS(M1)	-261161.282920	-0.008	-0.006	-0.005	-0.002	0.380	0.001	0.000	0.112	0.038
Ag, BS(M2)	-261161.282898	-0.008	-0.006	-0.005	-0.002	0.380	0.000	0.000	0.112	0.038

State	Energy / eV	Pr								
		s	p_z	p_x	p_y	$d(z^2)$	d(xz)	d(yz)	$d(x^2–y^2)$	d(xy)
Ag, HS	-690256.6194200	-0.008	-0.002	-0.004	0.008	0.010	0.004	0.000	0.409	0.064
Ag, BS(Ag)	-690256.6271823	0.009	0.002	0.003	-0.008	-0.010	-0.004	0.000	-0.405	-0.063
Ag, BS(M1)	-690256.6233938	-0.008	-0.002	-0.003	0.008	0.010	0.004	0.000	0.407	0.064
Ag, BS(M2)	-690256.6234035	-0.008	-0.002	-0.003	0.008	0.010	0.004	0.000	0.407	0.064

State	Energy / eV	Nd								
		s	p_z	p_x	p_y	$d(z^2)$	d(xz)	d(yz)	$d(x^2–y^2)$	d(xy)
Ag, HS	-712038.7522503	-0.008	-0.002	-0.003	0.008	0.010	0.004	0.000	0.408	0.066
Ag, BS(Ag)	-712038.7539078	0.008	0.002	0.003	-0.008	-0.010	-0.004	0.000	-0.408	-0.066
Ag, BS(M1)	-712038.7546793	-0.008	-0.002	-0.003	0.008	0.010	0.004	0.000	0.408	0.066
Ag, BS(M2)	-712038.7516820	-0.008	-0.002	-0.003	0.008	0.010	0.004	0.000	0.408	0.066

State	Energy / eV	Pm								
		s	p_z	p_x	p_y	$d(z^2)$	d(xz)	d(yz)	$d(x^2–y^2)$	d(xy)
Ag, HS	-734407.1112579	-0.008	-0.002	-0.004	0.008	0.010	0.004	0.000	0.408	0.064
Ag, BS(Ag)	-734407.1122200	0.009	0.002	0.003	-0.008	-0.010	-0.004	0.000	-0.407	-0.064
Ag, BS(M1)	-734407.1120134	-0.009	-0.002	-0.003	0.008	0.010	0.004	0.000	0.407	0.064
Ag, BS(M2)	-734407.1120158	-0.009	-0.002	-0.003	0.008	0.010	0.004	0.000	0.408	0.064

State	Energy / eV	Sm								
		s	p_z	p_x	p_y	$d(z^2)$	d(xz)	d(yz)	$d(x^2–y^2)$	d(xy)
Ag, HS	-757370.8307209	-0.009	-0.002	-0.004	0.008	0.010	0.004	0.001	0.398	0.072
Ag, BS(Ag)	-757370.8294215	0.009	0.002	0.003	-0.009	-0.010	-0.004	0.000	-0.398	-0.072
Ag, BS(M1)	-757370.8303880	-0.009	-0.002	-0.003	0.009	0.010	0.004	0.001	0.398	0.072
Ag, BS(M2)	-757370.8300333	-0.009	-0.002	-0.003	0.009	0.010	0.004	0.000	0.398	0.072

State	Energy / eV	Eu								
		s	p_z	p_x	p_y	$d(z^2)$	d(xz)	d(yz)	$d(x^2–y^2)$	d(xy)
Ag, HS	-780941.6451370	-0.009	-0.002	-0.004	0.009	0.010	0.004	0.000	0.405	0.063
Ag, BS(Ag)	-780941.6429831	0.009	0.002	0.003	-0.009	-0.010	-0.004	0.000	-0.405	-0.063
Ag, BS(M1)	-780941.6442593	-0.009	-0.002	-0.003	0.009	0.010	0.004	0.000	0.405	0.063
Ag, BS(M2)	-780941.6442483	-0.009	-0.002	-0.003	0.009	0.010	0.004	0.000	0.405	0.063

State	Energy / eV	Gd								
		s	p_z	p_x	p_y	$d(z^2)$	d(xz)	d(yz)	$d(x^2–y^2)$	d(xy)
Ag, HS	-805132.9916182	-0.010	-0.002	-0.003	0.011	0.010	0.004	0.000	0.405	0.062
Ag, BS(Ag)	-805132.9897960	0.010	0.002	0.003	-0.010	-0.010	-0.004	0.000	-0.405	-0.062
Ag, BS(M1)	-805132.9909971	-0.010	-0.002	-0.003	0.010	0.010	0.004	0.000	0.405	0.062
Ag, BS(M2)	-805132.9909952	-0.010	-0.002	-0.003	0.011	0.010	0.004	0.000	0.405	0.062

State	Energy / eV	Tb								
		s	p_z	p_x	p_y	$d(z^2)$	d(xz)	d(yz)	$d(x^2–y^2)$	d(xy)
Ag, HS	-829933.1530550	-0.009	-0.002	-0.003	0.010	0.011	0.004	0.000	0.393	0.072
Ag, BS(Ag)	-829933.1494068	0.009	0.002	0.003	-0.010	-0.011	-0.004	0.000	-0.394	-0.072
Ag, BS(M1)	-829933.1524994	-0.009	-0.002	-0.003	0.010	0.010	0.004	0.000	0.393	0.072
Ag, BS(M2)	-829933.1503849	-0.009	-0.002	-0.003	0.010	0.011	0.004	0.000	0.394	0.072

State	Energy / eV	Dy								
		s	p_z	p_x	p_y	$d(z^2)$	d(xz)	d(yz)	$d(x^2–y^2)$	d(xy)
Ag, HS	-855374.6983829	-0.009	-0.002	-0.003	0.011	0.011	0.004	0.000	0.403	0.062
Ag, BS(Ag)	-855374.6941362	0.009	0.002	0.003	-0.011	-0.011	-0.004	0.000	-0.403	-0.062
Ag, BS(M1)	-855374.6963800	-0.009	-0.002	-0.003	0.011	0.011	0.004	0.000	0.403	0.062
Ag, BS(M2)	-855374.6963801	-0.009	-0.002	-0.003	0.011	0.011	0.004	0.000	0.403	0.062

State	Energy / eV	Ho								
		s	p_z	p_x	p_y	$d(z^2)$	d(xz)	d(yz)	$d(x^2–y^2)$	d(xy)
Ag, HS	-881464.3917648	-0.009	-0.002	-0.003	0.011	0.011	0.004	0.000	0.402	0.062
Ag, BS(Ag)	-881464.3908812	0.008	0.002	0.003	-0.011	-0.011	-0.004	0.000	-0.402	-0.062
Ag, BS(M1)	-881464.3913977	-0.008	-0.002	-0.003	0.011	0.011	0.004	0.000	0.402	0.062
Ag, BS(M2)	-881464.3913346	-0.008	-0.002	-0.003	0.011	0.011	0.004	0.000	0.402	0.062

5. Fluoride [AgM_2F_8] clusters

Table S11. Total energy and Löwdin spin populations of each orbital (specified with m_l number) in 3d or 4f subshell for each 3d TM or Ln cation in the clusters of [AgM_2F_8] type.

State	Energy / eV	Co1					Co2				
		d(z^2)	d(xz)	d(yz)	d(x^2–y^2)	d(xy)	d(z^2)	d(xz)	d(yz)	d(x^2–y^2)	d(xy)
Cd, BS	-251932.535205	-0.88	-0.08	-0.45	-0.85	-0.47	0.88	0.08	0.42	0.83	0.50
Cd, HS	-251932.525115	0.88	0.08	0.45	0.85	0.47	0.88	0.08	0.42	0.83	0.50
Ag, HS	-244316.366165	0.87	0.10	0.46	0.86	0.46	0.88	0.10	0.43	0.84	0.49
Ag, BS(Ag)	-244316.350967	0.88	0.08	0.45	0.85	0.46	0.88	0.08	0.43	0.83	0.50
Ag, BS(M1)	-244316.371471	-0.88	-0.08	-0.45	-0.85	-0.47	0.88	0.10	0.43	0.84	0.49
Ag, BS(M2)	-244316.364831	0.87	0.10	0.45	0.86	0.46	-0.88	-0.08	-0.42	-0.83	-0.50

State	Energy / eV	Ni1					Ni2				
		d(z^2)	d(xz)	d(yz)	d(x^2–y^2)	d(xy)	d(z^2)	d(xz)	d(yz)	d(x^2–y^2)	d(xy)
Cd, BS	-258914.678628	-0.42	-0.02	-0.44	-0.57	-0.32	0.39	0.10	0.38	0.68	0.22
Cd, HS	-258914.675880	0.41	0.02	0.44	0.57	0.32	0.39	0.10	0.39	0.68	0.22
Ag, HS	-251298.501134	0.41	0.02	0.45	0.57	0.32	0.38	0.09	0.40	0.68	0.21
Ag, BS(Ag)	-251298.512098	0.41	0.02	0.45	0.57	0.31	0.38	0.10	0.39	0.68	0.22
Ag, BS(M1)	-251298.509556	-0.41	-0.02	-0.44	-0.57	-0.31	0.39	0.09	0.39	0.68	0.21
Ag, BS(M2)	-251298.509841	0.41	0.02	0.44	0.57	0.32	-0.38	-0.10	-0.39	-0.68	-0.22

State	Energy / eV	Cu1					Cu2				
		d(z^2)	d(xz)	d(yz)	d(x^2–y^2)	d(xy)	d(z^2)	d(xz)	d(yz)	d(x^2–y^2)	d(xy)
Cd, BS	-266275.939785	-0.45	0.00	-0.05	-0.11	-0.12	0.46	0.01	0.04	0.11	0.12
Cd, HS	-266275.934559	0.45	0.00	0.05	0.11	0.12	0.46	0.01	0.04	0.11	0.12
Ag, HS	-258659.782771	0.45	0.00	0.05	0.11	0.12	0.46	0.01	0.04	0.11	0.12
Ag, BS(Ag)	-258659.818013	0.45	0.00	0.05	0.11	0.12	0.45	0.01	0.04	0.11	0.12
Ag, BS(M1)	-258659.807810	-0.45	0.00	-0.05	-0.11	-0.12	0.46	0.01	0.04	0.11	0.12
Ag, BS(M2)	-258659.808730	0.45	0.00	0.05	0.11	0.12	-0.45	-0.01	-0.04	-0.11	-0.12

State	Energy / eV	Nd1							Nd2						
		f0	f+1	f-1	f+2	f-2	f+3	f-3	f0	f+1	f-1	f+2	f-2	f+3	f-3
Cd, BS	-709792.8836851	-0.29	-0.61	-0.35	-0.11	-0.71	-0.77	-0.14	0.28	0.81	0.16	0.79	0.07	0.06	0.81
Cd, HS	-709792.8835880	0.29	0.61	0.35	0.11	0.71	0.77	0.14	0.28	0.81	0.16	0.79	0.07	0.06	0.82
Ag, HS	-702176.6803322	0.29	0.61	0.35	0.11	0.71	0.77	0.14	0.28	0.81	0.16	0.78	0.07	0.06	0.81
Ag, BS(Ag)	-702176.6838464	0.28	0.62	0.36	0.11	0.70	0.78	0.13	0.28	0.81	0.16	0.78	0.07	0.06	0.82
Ag, BS(M1)	-702176.6840387	-0.28	-0.62	-0.36	-0.11	-0.70	-0.78	-0.13	0.28	0.81	0.16	0.78	0.07	0.06	0.81
Ag, BS(M2)	-702176.6802646	0.28	0.62	0.36	0.11	0.70	0.78	0.13	-0.25	-0.84	-0.18	-0.77	-0.06	-0.05	-0.83

State	Energy / eV	Pm1							Pm2						
		f0	f+1	f-1	f+2	f-2	f+3	f-3	f0	f+1	f-1	f+2	f-2	f+3	f-3
Cd, BS	-732161.2758065	-0.71	-0.63	-0.27	-0.87	-0.37	-0.95	-0.18	0.70	0.37	0.54	0.84	0.39	0.21	0.92
Cd, HS	-732161.2755478	0.71	0.63	0.28	0.87	0.37	0.94	0.18	0.70	0.37	0.54	0.84	0.39	0.21	0.92
Ag, HS	-724545.0576993	0.71	0.63	0.28	0.87	0.36	0.94	0.18	0.70	0.37	0.54	0.84	0.39	0.21	0.92
Ag, BS(Ag)	-724545.0585766	0.71	0.62	0.28	0.87	0.36	0.94	0.19	0.70	0.37	0.54	0.84	0.39	0.20	0.92
Ag, BS(M1)	-724545.0584061	-0.71	-0.62	-0.28	-0.88	-0.36	-0.93	-0.19	0.70	0.37	0.54	0.84	0.38	0.21	0.92
Ag, BS(M2)	-724545.0584184	0.71	0.63	0.28	0.87	0.37	0.94	0.18	-0.70	-0.37	-0.55	-0.84	-0.39	-0.20	-0.93

State	Energy / eV	Sm1							Sm2						
		f0	f+1	f-1	f+2	f-2	f+3	f-3	f0	f+1	f-1	f+2	f-2	f+3	f-3
Cd, BS	-755125.0453333	-0.95	-0.60	-0.12	-0.95	-0.97	-0.65	-0.72	0.95	0.54	0.21	0.94	0.97	0.54	0.83
Cd, HS	-755125.0450346	0.95	0.61	0.13	0.95	0.97	0.65	0.72	0.94	0.54	0.21	0.94	0.97	0.54	0.83
Ag, HS	-747508.8080837	0.95	0.61	0.13	0.95	0.97	0.65	0.72	0.94	0.54	0.21	0.94	0.97	0.54	0.83
Ag, BS(Ag)	-747508.8064226	0.95	0.61	0.12	0.95	0.97	0.64	0.73	0.95	0.54	0.21	0.94	0.97	0.54	0.83
Ag, BS(M1)	-747508.8075397	-0.95	-0.61	-0.12	-0.95	-0.97	-0.64	-0.73	0.95	0.54	0.21	0.94	0.97	0.54	0.83
Ag, BS(M2)	-747508.8075367	0.95	0.61	0.12	0.95	0.97	0.64	0.73	-0.95	-0.54	-0.21	-0.94	-0.97	-0.54	-0.83

Table S12. Total energy and Löwdin spin populations of each orbital (specified with m_l number) in 4*s*, 4*p* and 3*d* subshells for the silver cation in the clusters of [AgM_2F_8] type.

State	Energy / eV	Co								
		s	p_z	p_x	p_y	d(z^2)	d(xz)	d(yz)	d(x^2–y^2)	d(xy)
Ag, HS	-244316.366165	-0.021	-0.005	0.002	-0.003	0.370	0.001	0.013	0.159	0.000
Ag, BS(Ag)	-244316.350967	0.023	0.007	0.001	0.001	-0.383	0.000	-0.014	-0.168	0.000
Ag, BS(M1)	-244316.371471	-0.022	-0.006	0.001	-0.002	0.375	0.001	0.014	0.162	0.000
Ag, BS(M2)	-244316.364831	-0.022	-0.006	0.001	-0.002	0.378	0.000	0.014	0.164	0.000

State	Energy / eV	Ni								
		s	p_z	p_x	p_y	d(z^2)	d(xz)	d(yz)	d(x^2–y^2)	d(xy)
Ag, HS	-251298.501134	-0.018	-0.005	-0.001	-0.002	0.390	0.003	0.009	0.127	0.049
Ag, BS(Ag)	-251298.512098	0.018	0.007	0.001	0.001	-0.388	-0.002	-0.009	-0.126	-0.049
Ag, BS(M1)	-251298.509556	-0.018	-0.006	-0.001	-0.002	0.389	0.003	0.009	0.126	0.050
Ag, BS(M2)	-251298.509841	-0.018	-0.006	-0.001	-0.002	0.389	0.002	0.009	0.127	0.049

State	Energy / eV	Cu								
		s	p_z	p_x	p_y	d(z^2)	d(xz)	d(yz)	d(x^2–y^2)	d(xy)
Ag, HS	-258659.782771	-0.019	-0.006	-0.001	-0.002	0.401	0.000	0.003	0.169	0.000
Ag, BS(Ag)	-258659.818013	0.017	0.007	0.003	0.001	-0.396	0.000	-0.003	-0.165	0.000
Ag, BS(M1)	-258659.807810	-0.018	-0.006	-0.002	-0.002	0.398	0.000	0.003	0.167	0.000
Ag, BS(M2)	-258659.808730	-0.018	-0.006	-0.002	-0.002	0.398	0.000	0.003	0.167	0.000

State	Energy / eV	Nd								
		s	p_z	p_x	p_y	d(z^2)	d(xz)	d(yz)	d(x^2–y^2)	d(xy)
Ag, HS	-702176.6803322	-0.016	-0.002	-0.003	0.020	0.012	0.005	0.000	0.403	0.066
Ag, BS(Ag)	-702176.6838464	0.016	0.002	0.002	-0.020	-0.012	-0.005	0.000	-0.402	-0.065
Ag, BS(M1)	-702176.6840387	-0.016	-0.002	-0.002	0.020	0.012	0.005	0.000	0.402	0.065
Ag, BS(M2)	-702176.6802646	-0.016	-0.002	-0.002	0.020	0.012	0.005	0.000	0.403	0.066

State	Energy / eV	Pm								
		s	p_z	p_x	p_y	d(z^2)	d(xz)	d(yz)	d(x^2–y^2)	d(xy)
Ag, HS	-724545.0576993	-0.016	-0.002	-0.002	0.022	0.012	0.004	0.000	0.402	0.062
Ag, BS(Ag)	-724545.0585766	0.017	0.002	0.002	-0.022	-0.012	-0.004	0.000	-0.402	-0.062
Ag, BS(M1)	-724545.0584061	-0.016	-0.002	-0.002	0.022	0.012	0.004	0.000	0.402	0.062
Ag, BS(M2)	-724545.0584184	-0.016	-0.002	-0.002	0.022	0.012	0.004	0.000	0.402	0.062

State	Energy / eV	Sm								
		s	p_z	p_x	p_y	d(z^2)	d(xz)	d(yz)	d(x^2–y^2)	d(xy)
Ag, HS	-747508.8080837	-0.016	-0.002	-0.002	0.022	0.013	0.004	0.000	0.402	0.061
Ag, BS(Ag)	-747508.8064226	0.016	0.002	0.002	-0.023	-0.013	-0.004	0.000	-0.402	-0.061
Ag, BS(M1)	-747508.8075397	-0.016	-0.002	-0.002	0.022	0.013	0.004	0.000	0.402	0.061
Ag, BS(M2)	-747508.8075367	-0.016	-0.002	-0.002	0.022	0.013	0.004	0.000	0.402	0.061

6. XYZ geometries for selected clusters

XYZ cartesian coordinates provided in angstroms for all clusters with 3d metals and the following clusters with Ln: Eu/O^{2-}, Gd/O^{2-}, Ho/O^{2-}, Yb/O^{2-}, Pr/Cl^{-}, Sm/F^{-}, Sm/O^{2-} and Sm/Cl^{-}.

```
11
AgCo2F7Ominus1.xyz
  Ag      5.950540     5.991215     6.044541
  Co      8.415635     6.328347     4.112418
  Co      8.470004     5.679775     7.909705
  O       8.036140     6.002803     6.016673
  F       6.411167     5.611662     8.240284
  F       6.348086     6.373342     3.837018
  F       3.813023     5.980530     6.073304
  F       9.229692     4.966049     3.002974
  F       9.298935     7.049903     8.998327
  F       9.315320     4.033144     8.478564
  F       9.227435     7.983422     3.519711

11
AgCo2F8minus2.xyz
  Ag      6.017762     5.989951     6.052511
  Co      8.417012     6.363803     3.898178
  Co      8.455576     5.645806     8.109721
  F       6.441435     5.636763     8.115808
  F       6.386912     6.343913     3.983505
  F       3.994308     5.978378     6.096896
  F       9.118834     4.905856     2.967780
  F       8.223514     6.002397     6.005147
  F       9.171722     7.107824     9.017284
  F       9.184836     3.972047     8.485044
  F       9.104065     8.053454     3.501646

11
AgCo2F7Clminus2.xyz
  Ag      6.203718     5.991665     6.042627
  Co      8.324045     6.445441     3.431811
  Co      8.384661     5.563542     8.584443
  Cl      8.723137     6.003282     6.006562
  F       6.450220     5.635641     8.149059
  F       6.400720     6.351265     3.935020
  F       4.175980     5.980032     6.075063
  F       8.924032     4.999544     2.429354
  F       9.008799     7.009129     9.573009
  F       9.011028     3.872521     9.036848
  F       8.909636     8.148130     2.969724

11
AgCu2F7Ominus1.xyz
  Ag      5.857710     6.001231     6.041930
  Cu      8.411821     6.098476     4.151537
  Cu      8.456854     5.891922     7.874769
  F       6.395145     6.119674     8.199380
  F       6.330278     5.886507     3.871576
  F       3.717675     6.015984     6.073246
  F       8.798398     5.385488     2.434625
  O       7.920151     5.978842     6.017259
  F       8.876674     6.594727     9.588787
  F       9.913008     4.671071     7.804178
  F       9.838261     7.356267     4.176235

11
AgCu2F8minus2.xyz
  Ag      5.955950     6.001683     6.042652
  Cu      8.360001     6.142392     3.850354
  Cu      8.417739     5.861741     8.173254
  F       6.455326     6.158756     8.107900
  F       6.398708     5.844128     3.964068
  F       3.936497     6.002625     6.071078
  F       8.508814     5.408366     2.168897
  F       8.087870     6.000647     6.016499
  F       8.600991     6.561003     9.865515
  F       9.928519     4.856772     8.009729
  F       9.865558     7.162075     3.963578

11
AgNi2F7Ominus1.xyz
  Ag      5.991291     5.528459     6.048475
  Ni      8.383060     6.217710     4.094578
  Ni      8.451392     6.194617     7.924140
  F       6.533184     5.643794     8.276498
  F       6.446040     5.697227     3.804307
  F       3.927630     4.953372     6.081988
  F       8.774466     7.834202     3.109760
  O       8.007582     6.090118     6.015889
  F       8.832431     7.822291     8.890935
  F       9.648988     5.013086     8.874313
  F       9.519912     5.005314     3.112641

11
AgNi2F8minus2.xyz
  Ag      6.151895     5.574074     6.044552
  Ni      8.354744     6.378351     3.812214
  Ni      8.519709     6.045681     8.205243
  F       6.650593     5.383717     8.116413
  F       6.415287     5.985355     3.961493
  F       4.210902     5.021429     6.074324
  F       8.540828     7.853087     2.707176
  F       8.218192     6.163772     6.012599
  F       8.628481     7.847250     8.614454
  F       9.468322     4.871522     9.278187
  F       9.357024     4.875953     3.406868

11
AgNi2F7Clminus2.xyz
  Ag      6.433863     5.657481     6.043840
  Ni      8.270171     6.188024     3.138056
  Ni      8.377675     6.162580     8.881441
  Cl      8.821766     6.300706     6.001726
  F       6.629797     5.687637     8.200323
  F       6.547327     5.714884     3.880423
  F       4.479587     5.127329     6.077854
  F       8.247042     7.828315     2.316782
  F       8.390631     7.807087     9.693666
  F       9.220611     4.749195     9.691405
  F       9.097507     4.776954     2.308007
```

```
11
Ag-f06-Eu2F7O-minus1.xyz
  Ag      0.625216   -3.132445   -0.389102
  Eu      2.226206   -0.334432    0.405012
  Eu     -2.010194   -1.134117   -0.100889
  F      -3.018412   -0.808553    1.704828
  F       2.859795    1.075881   -1.006967
  F       1.033384   -5.029468   -0.811583
  F       2.768662   -2.419480   -0.053380
  O       0.183307   -1.082721    0.073021
  F      -2.770340    0.026868   -1.668771
  F      -1.645721   -3.250338   -0.592293
  F       2.599663    0.269454    2.374650
```

```
11
Ag-f07-Gd2F7O-minus1.xyz
  Ag      0.590992   -3.183169    0.008454
  Gd      2.229809   -0.312161    0.244909
  Gd     -1.984529   -1.115347   -0.268415
  F      -3.053627   -0.386777    1.369717
  F       2.959025    0.731033   -1.409565
  F       0.960170   -5.132902    0.023883
  F       2.737667   -2.437796    0.284527
  O       0.192110   -1.078525   -0.009189
  F      -2.647525   -0.326014   -2.083728
  F      -1.678960   -3.279433   -0.268125
  F       2.546433    0.701737    2.042055
```

```
11
Ag-f10-Ho2F7O-minus1.xyz
  Ag      0.588935   -3.186666    0.008003
  Ho      2.193445   -0.332192    0.237557
  Ho     -1.943191   -1.115086   -0.259251
  F      -2.996781   -0.374104    1.335724
  F       2.898155    0.719069   -1.375397
  F       0.955879   -5.135748    0.020638
  F       2.728393   -2.408285    0.269936
  O       0.193119   -1.086693   -0.004190
  F      -2.595243   -0.325589   -2.035306
  F      -1.685990   -3.243594   -0.260312
  F       2.514844    0.669536    1.997122
```

```
11
Ag-f13-Yb2F7O-minus1.xyz
  Ag      0.580290   -3.160457    0.008185
  Yb      2.168355   -0.344321    0.236332
  Yb     -1.914032   -1.108176   -0.259537
  F      -2.952264   -0.394290    1.315933
  F       2.883223    0.668759   -1.354954
  F       0.942323   -5.107832    0.023455
  F       2.726531   -2.373855    0.274360
  O       0.189018   -1.057965   -0.009048
  F      -2.555727   -0.357965   -2.018773
  F      -1.704643   -3.202876   -0.260672
  F       2.488490    0.619625    1.979243
```

```
11
Ag-f02-Pr2F7Cl-neutral.xyz
  Ag      0.564451   -3.056174    0.012784
  Pr      2.991985   -0.147337    0.335700
  Pr     -2.757973   -1.245243   -0.357482
  F      -4.051504   -0.866257    1.218440
  F       4.036170    0.648826   -1.269023
  F       0.934936   -5.000180    0.020592
  F       2.615016   -2.357462    0.260474
  Cl      0.061853   -0.420236    0.005373
  F      -3.618695   -0.807172   -2.192524
  F      -1.597927   -3.162574   -0.248085
  F       3.673252    0.594456    2.148275
```

```
11
Ag-f05-Sm2F7O-minus1.xyz
  Ag      0.663104   -3.075052   -0.626480
  Sm      2.208018   -0.359713    0.555899
  Sm     -2.019432   -1.168168   -0.052207
  F      -3.027709   -1.091399    1.799880
  F       2.777593    1.270309   -0.656401
  F       1.108048   -4.882528   -1.319458
  F       2.785476   -2.353713   -0.203230
  O       0.182709   -1.124876    0.119242
  F      -2.794754    0.191206   -1.467231
  F      -1.605940   -3.197645   -0.822787
  F       2.574451   -0.027775    2.607297
```

```
11
Ag-f05-Sm2F8-neutral.xyz
  Ag      0.635433   -3.434705    0.008438
  Sm      2.376139   -0.230876    0.258088
  Sm     -2.149482   -1.085587   -0.280068
  F      -3.141560   -0.375700    1.371004
  F       3.025007    0.775547   -1.409445
  F       1.000326   -5.381638    0.022732
  F       2.531515   -2.463020    0.244067
  F       0.145696   -0.833725   -0.003478
  F      -2.723888   -0.300311   -2.087719
  F      -1.482444   -3.220787   -0.242038
  F       2.634822    0.731449    2.052942
```

```
11
Ag-f05-Sm2F7Cl-neutral.xyz
  Ag      0.563335   -3.057030   -0.001130
  Sm      2.883711   -0.132442    0.305691
  Sm     -2.713277   -1.277201   -0.345365
  F      -3.887093   -0.814130    1.266266
  F       4.011922    0.617886   -1.220376
  F       0.954025   -4.995554    0.026833
  F       2.595378   -2.315633    0.238938
  Cl      0.016210   -0.413628   -0.046040
  F      -3.510702   -0.789317   -2.166236
  F      -1.600863   -3.171986   -0.239344
  F       3.538918    0.529680    2.115287
```

XYZ cartesian coordinates provided in angstroms for clusters with a neutral TEMPO ligand.

```
33
TEMPO_Gd2F5-plus1
  Gd     0.981224    -1.570426    1.920718
  Gd    -1.258610    -2.079848   -1.191899
  F     -0.217943    -2.926652    0.603399
  F      2.992633    -1.793190    1.882427
  F      0.120203    -0.582671    3.460688
  F     -3.277081    -2.002683   -1.105443
  F     -0.357114    -1.869202   -2.991837
  O     -0.020630    -0.297871    0.029731
  C     -1.084127     1.806870   -0.658124
  C      1.420966     1.671907   -0.250002
  C      0.959025     3.131456   -0.434401
  C     -0.363087     3.050920   -1.210984
  H      0.805209     3.597742    0.540228
  H      1.714168     3.712724   -0.961248
  H     -0.973830     3.943308   -1.081772
  H     -0.178275     2.929179   -2.279978
  N      0.097677     0.969306   -0.275604
  C     -1.892032     2.110432    0.617628
  H     -2.767369     2.700104    0.341734
  H     -2.239430     1.193548    1.095193
  H     -1.311803     2.680936    1.343028
  C     -1.945312     1.087469   -1.695562
  H     -2.575485     0.311038   -1.247722
  H     -2.649666     1.801316   -2.125814
  H     -1.350769     0.692955   -2.524366
  C      2.262717     1.164051   -1.434803
  H      3.231259     1.664445   -1.410371
  H      2.437502     0.089621   -1.369108
  H      1.790274     1.380263   -2.393444
  C      2.136789     1.429682    1.081326
  H      2.625467     0.450448    1.124101
  H      2.951511     2.149078    1.177460
  H      1.475690     1.581521    1.938628
```

```
33
TEMPO_Gd2F4O-neutral
  Gd     0.176791    -1.571689    2.232662
  Gd    -0.422691    -2.415712   -1.226461
  O      0.019832    -0.268693    0.055889
  N      0.120305     0.979002   -0.274396
  O     -0.013887    -2.998306    0.723524
  F     -1.536619    -1.103282    3.327440
  F      2.056611    -0.958692    2.922723
  F     -2.436465    -2.105145   -1.697834
  F      1.098165    -2.263059   -2.663790
  C     -1.088135     1.846054   -0.448195
  C      1.443550     1.641898   -0.509268
  C      1.012439     3.120215   -0.480316
  C     -0.438648     3.138747   -0.983601
  H      1.068821     3.495853    0.543589
  H      1.672202     3.734053   -1.092981
  H     -0.982508     4.019875   -0.644418
  H     -0.464736     3.137298   -2.074732
  C     -1.743085     2.036352    0.930214
  H     -2.631772     2.658770    0.815803
  H     -2.055029     1.082384    1.357748
  H     -1.069766     2.530863    1.632860
  C     -2.063177     1.207677   -1.439096
  H     -2.473552     0.265474   -1.076375
  H     -2.894084     1.896127   -1.603678
  H     -1.584351     1.028798   -2.404020
  C      1.965684     1.202921   -1.890225
  H      2.943026     1.660418   -2.053912
  H      2.080025     0.119561   -1.948653
  H      1.302671     1.521164   -2.696217
  C      2.438752     1.279663    0.589601
  H      2.709625     0.225365    0.570283
  H      3.348918     1.862568    0.436429
  H      2.054349     1.512716    1.583213
```

```
33
TEMPO_Ni2F5-minus1
  Ni    -0.336510    -1.163765    2.051294
  Ni     0.273402    -2.305475   -0.935963
  O     -0.185684    -0.273318   -0.123848
  N      0.007137     0.953399   -0.445424
  F      0.117318    -2.719907    0.984041
  F     -2.120600    -1.026508    2.431109
  F      0.923884    -0.544602    3.215719
  F     -0.778321    -3.388811   -1.955635
  F      1.997516    -1.903643   -1.474226
  C     -1.107564     1.935999   -0.340590
  C      1.361447     1.527599   -0.719643
  C      1.066677     3.028681   -0.523679
  C     -0.419110     3.203586   -0.878970
  H      1.240837     3.302811    0.519458
  H      1.719426     3.645872   -1.142986
  H     -0.850851     4.106364   -0.444423
  H     -0.546699     3.263266   -1.962840
  C     -1.536542     2.079693    1.129404
  H     -2.335420     2.822734    1.197907
  H     -1.914077     1.134046    1.524466
  H     -0.707202     2.407684    1.759263
  C     -2.283196     1.453480   -1.192248
  H     -2.648146     0.496914   -0.817372
  H     -3.099168     2.178523   -1.145050
  H     -1.987742     1.328049   -2.235958
  C      1.770179     1.196204   -2.165194
  H      2.744415     1.647438   -2.372941
  H      1.859083     0.114424   -2.283707
  H      1.050420     1.589664   -2.887657
  C      2.387357     0.960716    0.262625
  H      2.565949    -0.093416    0.048131
  H      3.328712     1.503990    0.140457
  H      2.056337     1.057545    1.298293
```

7. SI References

[1] C. Benelli, D. Gatteschi, *Chem. Rev.* **2002**, *102*, 2369–2388.

[2] F. Gendron, J. Autschbach, J.-P. Malrieu, H. Bolvin, *Inorg. Chem.* **2019**, *58*, 581–593.

[3] S.-H. Jang, R. Sano, Y. Kato, Y. Motome, *Phys. Rev. Materials* **2020**, *4*, 104420.

[4] J. Long, F. Habib, P.-H. Lin, I. Korobkov, G. Enright, L. Ungur, W. Wernsdorfer, L. F. Chibotaru, M. Murugesu, *J. Am. Chem. Soc.* **2011**, *133*, 5319–5328.

[5] L. Peters, S. Ghosh, B. Sanyal, C. van Dijk, J. Bowlan, W. de Heer, A. Delin, I. Di Marco, O. Eriksson, M. I. Katsnelson, B. Johansson, A. Kirilyuk, *Sci Rep* **2016**, *6*, 19676.

[6] M. L. Kahn, R. Ballou, P. Porcher, O. Kahn†, J.-P. Sutter, *Chemistry – A European Journal* **2002**, *8*, 525–531.

[7] T. Nakamura, T. Kanetomo, T. Ishida, *Inorg. Chem.* **2021**, *60*, 535–539.

[8] I. A. Lutsenko, M. A. Kiskin, S. A. Nikolaevskii, A. A. Starikova, N. N. Efimov, A. V. Khoroshilov, A. S. Bogomyakov, I. V. Ananyev, J. K. Voronina, A. S. Goloveshkin, A. A. Sidorov, I. L. Eremenko, *ChemistrySelect* **2019**, *4*, 14261–14270.

[9] G. Rajaraman, F. Totti, A. Bencini, A. Caneschi, R. Sessoli, D. Gatteschi, *Dalton Transactions* **2009**, *0*, 3153–3161.

[10] M. K. Singh, T. Rajeshkumar, R. Kumar, S. K. Singh, G. Rajaraman, *Inorg. Chem.* **2018**, *57*, 1846–1858.

[11] D.-P. Liu, X.-P. Lin, H. Zhang, X.-Y. Zheng, G.-L. Zhuang, X.-J. Kong, L.-S. Long, L.-S. Zheng, *Angewandte Chemie* **2016**, *128*, 4608–4612.

[12] L. E. Sweet, L. E. Roy, F. Meng, T. Hughbanks, *J. Am. Chem. Soc.* **2006**, *128*, 10193–10201.

[13] O. V. Yazyev, L. Helm, V. G. Malkin, O. L. Malkina, *J. Phys. Chem. A* **2005**, *109*, 10997–11005.

[14] R. Modak, Y. Sikdar, A. Bieńko, M. Witwicki, M. Jerzykiewicz, S. Goswami, *Polyhedron* **2016**, *119*, 202–215.

[15] F. Neese, *Wiley Interdisciplinary Reviews: Computational Molecular Science* **2012**, *2*, 73–78.

[16] A. D. Becke, *J. Chem. Phys.* **1993**, *98*, 5648–5652.

[17] C. Lee, W. Yang, R. G. Parr, *Phys. Rev. B* **1988**, *37*, 785–789.

[18] E. van Lenthe, E. J. Baerends, J. G. Snijders, *J. Chem. Phys.* **1994**, *101*, 9783–9792.

[19] C. van Wüllen, *J. Chem. Phys.* **1998**, *109*, 392–399.

[20] J. D. Rolfes, F. Neese, D. A. Pantazis, *Journal of Computational Chemistry* **2020**, *41*, 1842–1849.

[21] D. A. Pantazis, F. Neese, *J. Chem. Theory Comput.* **2009**, *5*, 2229–2238.

[22] X. Cao, M. Dolg, *J. Chem. Phys.* **2001**, *115*, 7348–7355.

[23] B. O. Roos, R. Lindh, P.-Å. Malmqvist, V. Veryazov, P.-O. Widmark, A. C. Borin, *J. Phys. Chem. A* **2008**, *112*, 11431–11435.

[24] C. Adamo, P. Maldivi, *J. Phys. Chem. A* **1998**, *102*, 6812–6820.

[25] L. Noodleman, *J. Chem. Phys.* **1981**, *74*, 5737–5743.

[26] P.-O. Löwdin, *Phys. Rev.* **1955**, *97*, 1474–1489.

[27] F. He, M.-L. Tong, X.-M. Chen, *Inorg. Chem.* **2005**, *44*, 8285–8292.

[28] J.-P. Costes, F. Dahan, A. Dupuis, J.-P. Laurent, *New J. Chem.* **1998**, *22*, 1525–1529.

[29] G. Novitchi, S. Shova, A. Caneschi, J.-P. Costes, M. Gdaniec, N. Stanica, *Dalton Trans.* **2004**, 1194–1200.
